# Do general and specific factors of preschool psychopathology predict preadolescent outcomes? A transdiagnostic hierarchical approach

**DOI:** 10.1017/S003329172200246X

**Published:** 2022-08-24

**Authors:** Giorgia Michelini, Kelly Gair, Yuan Tian, Jiaju Miao, Lea R. Dougherty, Brandon L. Goldstein, Leigha A. MacNeill, Deanna M. Barch, Joan L. Luby, Lauren S. Wakschlag, Daniel N. Klein, Roman Kotov

**Affiliations:** 1 Department of Biological & Experimental Psychology, School of Biological & Behavioural Sciences, Queen Mary University of London, London, UK;; 2 Semel Institute for Neuroscience & Human Behavior, University of California Los Angeles, Los Angeles, CA, USA;; 3 Department of Psychology, Stony Brook University, Stony Brook, NY, USA;; 4 Department of Applied Mathematics and Statistics, Stony Brook University, Stony Brook, NY, USA;; 5 Department of Psychology, University of Maryland, College Park, MD, USA;; 6 Department of Psychiatry, University of Connecticut School of Medicine, Farmington, CT, USA;; 7 Department of Medical Social Sciences, Feinberg School of Medicine, and Institute for Innovations in Developmental Sciences, Northwestern University, Chicago, IL, USA;; 8 Departments of Psychological & Brain Sciences, Washington University, St. Louis, MO, USA;; 9 Departments of Psychiatry, Washington University, St. Louis, MO, USA;; 10 Departments of Radiology, Washington University, St. Louis, MO, USA; 11 Department of Psychiatry & Behavioral Health, Stony Brook University, Stony Brook, NY, USA

**Keywords:** Preschool, preadolescence, p factor, transdiagnostic dimension, specific dimension, predictive utility

## Abstract

**Background.:**

Preschool psychiatric symptoms significantly increase the risk for long-term negative outcomes. Transdiagnostic hierarchical approaches that capture general (‘p’) and specific psychopathology dimensions are promising for understanding risk and predicting outcomes, but their predictive utility in young children is not well established. We delineated a hierarchical structure of preschool psychopathology dimensions and tested their ability to predict psychiatric disorders and functional impairment in preadolescence.

**Methods.:**

Data for 1253 preschool children (mean age = 4.17, s.d. = 0.81) were drawn from three longitudinal studies using a similar methodology (one community sample, two psychopathology-enriched samples) and followed up into preadolescence, yielding a large and diverse sample. Exploratory factor models derived a hierarchical structure of general and specific factors using symptoms from the Preschool Age Psychiatric Assessment interview. Longitudinal analyses examined the prospective associations of preschool *p* and specific factors with preadolescent psychiatric disorders and functional impairment.

**Results.:**

A hierarchical dimensional structure with a *p* factor at the top and up to six specific factors (distress, fear, separation anxiety, social anxiety, inattention-hyperactivity, oppositionality) emerged at preschool age. The *p* factor predicted all preadolescent disorders (Δ*R*^2^ = 0.04–0.15) and functional impairment (Δ*R*^2^ = 0.01–0.07) to a significantly greater extent than preschool psychiatric diagnoses and functioning. Specific dimensions provided additional predictive power for the majority of preadolescent outcomes (disorders: Δ*R*^2^ = 0.06–0.15; functional impairment: Δ*R*^2^ = 0.05–0.12).

**Conclusions.:**

Both general and specific dimensions of preschool psychopathology are useful for predicting clinical and functional outcomes almost a decade later. These findings highlight the value of transdiagnostic dimensions for predicting prognosis and as potential targets for early intervention and prevention.

## Introduction

Psychiatric symptoms are as common in preschoolers as they are in school-aged children (Dougherty et al., 2015; Egger & Angold, 2006). Emotional and behavioral difficulties in early childhood contribute to a negative cascade with long-lasting effects over development and prospectively predict future psychopathology at school age and beyond (Finsaas, Bufferd, Dougherty, Carlson, & Klein, 2018; Luby, Gaffrey, Tillman, April, & Belden, 2014). Assessing and monitoring the emergence of preschool symptoms is an important step for the design and implementation of prevention and early intervention efforts (Wakschlag et al., 2019). Yet, pragmatic methods for predicting the course of psychopathology from preschool age and informing the prevention of future psychiatric outcomes are still underdeveloped (Luby et al., 2019).

A challenge for clinical practice and research in preschool populations is that traditional psychiatric diagnoses are not tailored to preschool children and do not provide guidelines for diagnosing most conditions before school age (APA, 2013; WHO, 1992). General skepticism toward preschool diagnoses among clinicians often arises from concerns regarding ‘labeling’ young children with psychopathology, given the stigma around mental illness (Heflinger & Hinshaw, 2010) and the ‘they’ll grow out of it’ myth (Luby, 2012). Further, the methodological artifacts induced by binary categorical diagnoses often mean that preschool children may shift between above and below clinical cutoffs despite showing a relatively stable clinical picture (Angold, Costello, Farmer, Burns, & Erkanli, 1999). A promising approach to address these concerns, consistent with the Hierarchical Taxonomy of Psychopathology (HiTOP; Kotov et al., 2017) and Research Domain Criteria (RDoC; Cuthbert, 2014) frameworks, is to describe psychopathology using transdiagnostic dimensions, rather than binary diagnoses. Transdiagnostic dimensional approaches allow a finer-grained characterization of clinical problems across a continuum of severity (including subthreshold presentations) and better account for the widespread co-occurrence and heterotypic developmental continuity between different forms of psychopathology (Michelini, Palumbo, DeYoung, Latzman, & Kotov, 2021; Wakschlag et al., 2012). Moreover, they provide richer information for understanding risk and greater clinical utility than narrow band diagnoses at younger ages. This is in line with current clinical approaches toward the early prevention of psychopathology, which largely focus on self-regulation promotion via parent training (Forbes, Rapee, & Krueger, 2019; Wakschlag et al., 2019).

Among transdiagnostic approaches, hierarchical dimensional approaches consistent with the HiTOP framework delineate major latent dimensions of psychopathology organized across multiple hierarchical levels, from general to specific (Caspi et al., 2014; Kotov et al., 2017). Several studies using either exploratory factor analysis (EFA) and bifactor approaches in school-aged children and adults have identified a general psychopathology (‘*p*’) factor at the apex, which is thought to reflect a general liability to all mental health problems and accounts for their common co-occurrence (Allegrini et al., 2020; Caspi et al., 2014; Lahey et al., 2012). A number of specific dimensions have also been delineated with these approaches (e.g. fear, distress, antisocial behavior) (Achenbach, Ivanova, & Rescorla, 2017; Carragher et al., 2016; Clark et al., 2021; Forbes, Tackett, Markon, & Krueger, 2016; Michelini et al., 2019; St Clair et al., 2017), which may differ with age (Michelini et al., 2019). Several dimensional measures exist for assessing preschool psychopathology (Achenbach et al., 2017; Essex et al., 2002; Goodman, 2001), but empirical efforts for characterizing preschool psychopathology through latent transdiagnostic dimensions have been very limited (Sterba, Egger, & Angold, 2007; Wichstrøm & Berg-Nielsen, 2014), and none have characterized it hierarchically in a sufficiently detailed fashion. A few available preschool studies have applied confirmatory bifactor models, including a general *p* factor alongside specific internalizing and externalizing factors (Olino, Dougherty, Bufferd, Carlson, & Klein, 2014; Olino et al., 2018) or internalizing, externalizing and inattention-hyperactivity factors (McElroy, Belsky, Carragher, Fearon, & Patalay, 2018). Yet, because these findings are based on pre-specified confirmatory models, it is unclear whether a greater number of specific dimensions can be identified in preschoolers, in line with EFA studies of older children and adults (Carragher et al., 2016; Forbes et al., 2016, 2021a, 2021b; Kim & Eaton, 2015; Michelini et al., 2019).

Furthermore, little is known about the effects of preschool *p* factor and specific dimensions of psychopathology on future psychiatric disorders and functional impairment. Given the widespread heterotypic continuity commonly observed across development (Caspi et al., 2020; Finsaas et al., 2018), conceptualizing risk for future psychopathology and associated functional outcomes with a *p* factor at preschool age may be more useful for predicting these outcomes than using traditional diagnostic categories (Pettersson, Lahey, Larsson, & Lichtenstein, 2018). Further, previous evidence in middle childhood shows that specific dimensions may explain certain clinical and functional outcomes over and above the *p* factor (Michelini et al., 2019). Investigating the ability of preschool general and specific dimensions to predict future outcomes can help establish their clinical utility and potentially inform future prognostic and preventive efforts.

The current study aimed to delineate a hierarchical structure of preschool psychopathology dimensions and test their ability to predict psychiatric and functional outcomes in preadolescence. We aggregated data from three longitudinal samples of preschool children that utilized the same clinical interview of preschool psychopathology and were followed up into preadolescence, yielding a large, socio-demographically and racially diverse sample. We chose a follow-up time point in preadolescence based on the availability of preadolescent assessments in all three samples and because the onset of psychiatric disorders is common in this developmental period (Kessler et al., 2005). We tested the alternative hypotheses that (a) the preschool *p* factor alone would significantly predict preadolescent psychiatric disorders and functional impairment, without a further increase in predictive power when also considering specific dimensions; or that (b) specific dimensions would enhance predictive power beyond the *p* factor.

## Methods

### Sample

The sample consisted of participants between 3 and 6 years followed up into preadolescence and drawn from three naturalistic longitudinal studies with a number of similar characteristics (Table 1): the Multidimensional Assessment of Preschoolers Study (MAPS, *N* = 410; Wakschlag et al., 2012; Wiggins, Briggs-Gowan, Brotman, Leibenluft, & Wakschlag, 2021), the Preschool Depression Study (PDS, *N* = 302; Gaffrey, Tillman, Barch, & Luby, 2018; Luby, Si, Belden, Tandon, & Spitznagel, 2009), and the Stony Brook Temperament Study (SBTS, *N* = 541; Klein & Finsaas, 2017). In all three samples, parents provided written informed consent and all study procedures were approved by local Institutional Review Boards at Northwestern University, Washington University School of Medicine, and Stony Brook University (Klein & Finsaas, 2017; Luby et al., 2009; Wakschlag et al., 2012).

MAPS is a sample enriched for psychopathology which was recruited from multiple pediatric clinics within the Chicago, Illinois area (Wakschlag et al., 2012; Wiggins et al., 2021). Parents with young children from a larger unselected sample were invited to participate, oversampling for child disruptive behavior (above the 80th percentile on the Multidimensional Assessment Profile of Disruptive Behavior) (Wakschlag et al., 2012) and parental intimate partner violence (mother-reported past-year intimate partner violence). Eligibility criteria were English-speaking biological mother and absence of developmental delays. Of 425 children in this clinical subsample, 49.9% were Black, 29.9% were Hispanic, 18.6% were White, and 1.6% belonged to other racial/ethnical groups. Parental respondents were mainly biological mothers (91%), with 50% of all parents being married. Just under half (49.2%) of the participant families were from a poor background (Wakschlag et al., 2012).

PDS is a sample enriched for preschool psychopathology via oversampling of child depressive symptoms. Participants from the PDS were recruited near St. Louis, Missouri, as part of a longitudinal study of preschool depression (Gaffrey et al., 2018; Luby et al., 2009). Recruitment was done through primary care practices, and preschools/daycares that were accessible to the general community in order to increase the socioeconomic and ethnic diversity of the sample. Recruitment sites were chosen at random using a geographically stratified method. Initial recruitment involved 1474 families, with a child between 3 and 6 years old, screened with the Preschool Feelings Checklist to assess depressive symptoms (Luby, Heffelfinger, Mrakotsky, & Hildebrand, 1999). Preschoolers with endorsed depressive symptoms (scores >3, i.e. above established cut-off) and with no depressive symptoms (scores of 0, i.e. healthy controls) were invited to join the study. A total of 305 preschoolers aged 3–6 years old completed the baseline assessment. The primary reporting caregivers were predominately biological mothers (92%). About half of the children (53.9%) were White and 100 (32.9%) were Black. Among participating parents, 130 (43.4%) had at least a four-year college degree.

SBTS is an unselected community sample of preschool children (Klein & Finsaas, 2017). Participants within a 20-mile radius of a suburban community in Stony Brook, New York, were recruited from a commercial mailing list as part of a longitudinal study examining temperament and risk for psychopathology (Klein & Finsaas, 2017; Olino et al., 2014). Families with a 3-year-old child and an English-speaking biological parent were invited to participate. Exclusion criteria were limited to significant medical conditions or developmental disabilities. Out of the 815 families who were invited to participate, 66.4% agreed to enter the study, leaving a final sample of 559 families. One biological parent per family (98.5% mothers) reported on their child’s psychopathology symptoms. Informed consent was obtained from the parent prior to participation. Most children were White (94.5%) and non-Hispanic (90.8%). Most of the parents in the sample were married or cohabiting (96%), just over half of the parents (55% of the mothers, 47.1% of the fathers) had at least a four-year college degree, and the median household income was between $70 000 and 90 000. These socio-demographic characteristics, although not representative of the entire U.S. population, are representative of the community from which the sample was drawn (Bufferd, Dougherty, Carlson, & Klein, 2011).

The total sample included 1253 children (mean age = 4.24, s.d. = 0.64; Table 1), of which 48% were girls and 60% were White. All three studies included follow-up assessments when participants were aged 9–12 years (mean age = 11.06, s.d. = 0.72). The current analysis focused on 410 participants from MAPS, 302 participants from PDS, and 541 participants from SBTS with data on the PAPA (Egger et al., 2006) in young childhood. In preadolescence, 298 (70%) of MAPS participants, 235 (77%) of PDS participants, and 434 (80%) of SBTS participants completed assessments of psychiatric diagnoses and functioning. There were no differences in socio-demographic characteristics between participants who did and did not complete preadolescent follow-up assessments in MAPS (age: *t* = −0.12, *p* = 0.90; sex: χ^2^ = 2.03, *p* = 0.15; race/ethnicity: χ^2^ = 4.27, *p* = 0.11; poor background: χ^2^ = 0.58, *p* = 0.45) and PDS (Gaffrey et al., 2018). SBTS children who completed follow-up assessments were slightly younger than those who were lost at follow-up, but the two groups did not differ on any other socio-demographic characteristics (Finsaas et al., 2018). Demographic characteristics and rates of psychiatric diagnoses at preschool and preadolescent assessments are given in Table 1.

### Measures

#### Preschool assessments

Across all three study sites, participating parents were administered the Preschool Age Psychiatric Assessment (PAPA) by trained interviewers (Egger et al., 2006). The PAPA is a structured diagnostic interview designed to assess common parent-reported DSM-IV psychiatric disorders in preschoolers in the 3 months before the interview. It covers a comprehensive set of DSM-IV symptoms using questions eliciting developmentally appropriate examples of symptom presentation. From the PAPA, we used individual symptoms for modeling psychopathology dimensions. Since the full PAPA interview was administered in the three samples with small modifications, we first harmonized the data across the three samples to obtain a common set of symptoms (see online Supplementary Material). We also created a composite of PAPA incapacity ratings (withdrawal and discord ratings) across domains of functioning to obtain a global index of preschool functional impairment.

#### Preadolescent assessments

DSM-IV psychiatric diagnoses were assessed at the preadolescent follow-up assessments using structured interviews: the Kiddie Schedule for Affective Disorders and Schizophrenia for School-Age Children Present and Lifetime (K-SADS-PL; Kaufman et al., 1997) in SBTS and MAPS, and the Child and Adolescent Psychiatric Assessment (CAPA; Angold et al., 1995) in PDS. Interviews were conducted with participating parents by trained interviewers at all study sites. In SBTS, interviews were also conducted with preadolescent participants, and reports were combined by the interviewer to assign diagnostic status. Our analyses focused on a subset of preadolescent disorders that were assessed in all three samples, namely depressive disorder (major depressive disorder or dysthymia), generalized anxiety disorder (GAD), separation anxiety disorder, attention-deficit/hyperactivity disorder (ADHD), oppositional defiant disorder (ODD), and conduct disorder (CD) in MAPS and PDS. Current diagnoses at the preadolescent follow-up were used in MAPS and PDS, which allowed greater comparability between diagnoses assessed in these samples. In SBTS, due to the low rates of current diagnoses at preadolescence in this population-based sample, we used diagnoses met in the interval between two assessments carried out at age 9 and at age 12 (i.e. interval diagnoses). This yielded a rate of diagnoses that was more comparable to rates in MAPS and PDS (Table 1).

Functional impairment was measured using the total functioning score from the parent-rated MacArthur Health and Behavior Questionnaire (HBQ; Essex et al., 2002) in MAPS and PDS, and the interviewer-rated Children’s Global Assessment Scale (CGAS; Shaffer et al., 1983) in SBTS.

### Statistical analysis

Analyses involved two steps. First, we explicated the hierarchical structure of preschool psychopathology through a latent dimensional approach based on EFA. To increase statistical power and achieve accurate model estimates (Conway, Forbes, & South, 2022), the three samples were combined beforehand through data harmonization and preparation steps (online Supplementary Material). Second, longitudinal analyses tested the prospective association of preschool dimensions with preadolescent psychiatric diagnoses and functional impairment. MAPS and PDS were combined also for these prospective analyses (MAPS + PDS; total follow-up *N* = 533), whereas SBTS was analyzed separately (follow-up *N* = 434), due to the aforementioned differences in the available preadolescent measures (i.e. current diagnoses and HBQ functioning in MAPS + PDS; interval diagnoses and CGAS functioning in SBTS).

#### Factor analysis on preschool symptoms of psychopathology

We examined the factor structure of the individual PAPA symptoms by extracting factor solutions with an increasing number of factors, using exploratory structural equation modeling (ESEM) in Mplus (Muthén and Muthén, Los Angeles, CA). Since we did not include model constraints, the results of this ESEM analysis are identical to those from a standard EFA. This exploratory approach was preferred over a confirmatory factor analysis as the number of dimensions and their composition across hierarchical levels in preschoolers was uncertain due to the paucity of relevant literature. The maximum number of factors was determined with parallel analyses, with extraction stopped when eigenvalues fell within the 95% confidence interval of eigenvalues from simulated data (Floyd & Widaman, 1995). Since the parallel analysis has a tendency to over-factor, we also examined the interpretability of factor solutions, defined as the presence of at least 4 primary loadings (highest loading ≥0.35 and at least 0.10 greater than all other loadings) for each factor (Fabrigar, Wegener, MacCallum, & Strahan, 1999; Velicer & Fava, 1998). Factors were rotated using an oblique rotation (goemin) to allow the extracted dimensions to correlate as expected (Kim & Eaton, 2015; Michelini et al., 2019). All factor structures from one to the maximum number of factors were considered.

To map the hierarchical structure and transitions between increasingly complex factor solutions, we correlated factor scores from models with an increasing number of factors, using Goldberg’s bass-ackwards method (Goldberg, 2006). We chose this approach as the primary method for extracting a hierarchy of psychopathology factors because, to our knowledge, it is the only available method to delineate multiple (i.e. >2) levels of a hierarchical structure from factors derived through exploratory factor models. Furthermore, this approach allowed us to compare our results with previous studies that used this method in older children and adults (Allegrini et al., 2020; Conway, Latzman, & Krueger, 2020; Forbes et al., 2017, 2021b; Kim & Eaton, 2015; Michelini et al., 2019; Tackett, Quilty, Sellbom, Rector, & Bagby, 2008; Wright et al., 2012). Factors in the derived hierarchical structure can be interpreted as interconnected across hierarchical levels, from a 1-factor level to narrower and more specific dimensions, allowing the examination of how different aspects of psychopathology shift and reorganize from one level to the next (Goldberg, 2006). As shown in several previous studies of psychopathology (and other constructs, e.g. personality and intelligence, or ‘g’), the general factor from this analytic approach is virtually identical to the general factor from confirmatory bifactor models (Clark et al., 2021; Forbes et al., 2021a; Fried, Greene, & Eaton, 2021; Kim & Eaton, 2015; Mansolf & Reise, 2017; Morgan, Hodge, Wells, & Watkins, 2015; Murray & Johnson, 2013; Murray, Booth, Eisner, Obsuth, & Ribeaud, 2019; van Bork, Epskamp, Rhemtulla, Borsboom, & van der Maas, 2017). Since general and specific factors of psychopathology have also previously been estimated using bifactor models (Caspi et al., 2014; Lahey et al., 2015, 2018; St Clair et al., 2017), we tested an additional confirmatory bifactor model. Each PAPA item was specified to load onto a general factor and one specific factor, based on its primary loading in the factor solution from exploratory models that we identified to include the maximum number of interpretable factors.

#### Prospective analyses of preschool psychopathology dimensions and preadolescent outcomes

We entered the following variables as consecutive blocks into hierarchical regressions: covariates (sex, preschool age; block 1), presence of any preschool DSM diagnosis (in models predicting preadolescent disorders) or preschool functioning (in models predicting preadolescent functional impairment) (block 2), *p* factor (block 3), and specific factors (block 4). Logistic regressions were used for psychiatric diagnoses (binary outcomes), whereas linear regressions were used for functional impairment (continuous outcomes). We examined the incremental predictive effects of each block by testing whether the change in *R*^2^ between blocks was significant, reflecting new information not captured in previous blocks. Area under the curve (AUC) from receiver operating characteristic analysis and AUC change are also reported for binary outcomes, but AUCs should be interpreted cautiously, especially for preadolescent disorders with low rates, such as CD (Table 1). Additional analyses in SBTS tested predictors of first-onset psychiatric disorders after removing children with preschool diagnoses. While our primary analyses focused on the *p* factor and specific factors from the solution with the maximum number of factors, an additional analysis examined the predictive power of intermediate factor solutions. Finally, we examined the predictive power of each dimension (*p* and specific factors) on its own, testing their associations with preadolescent outcomes while controlling for sex and preschool age. As a sensitivity analysis, all prospective analyses were also repeated in MAPS and PDS separately. Race and ethnicity were not included as covariates in prospective analyses because these variables reflect complex social constructs and their inclusion in prediction models may perpetrate disparities (MacNeill et al., 2021; Obermeyer, Powers, Vogeli, & Mullainathan, 2019).

## Results

### Hierarchical dimensional structure of preschool psychopathology

**Fig. 1.**
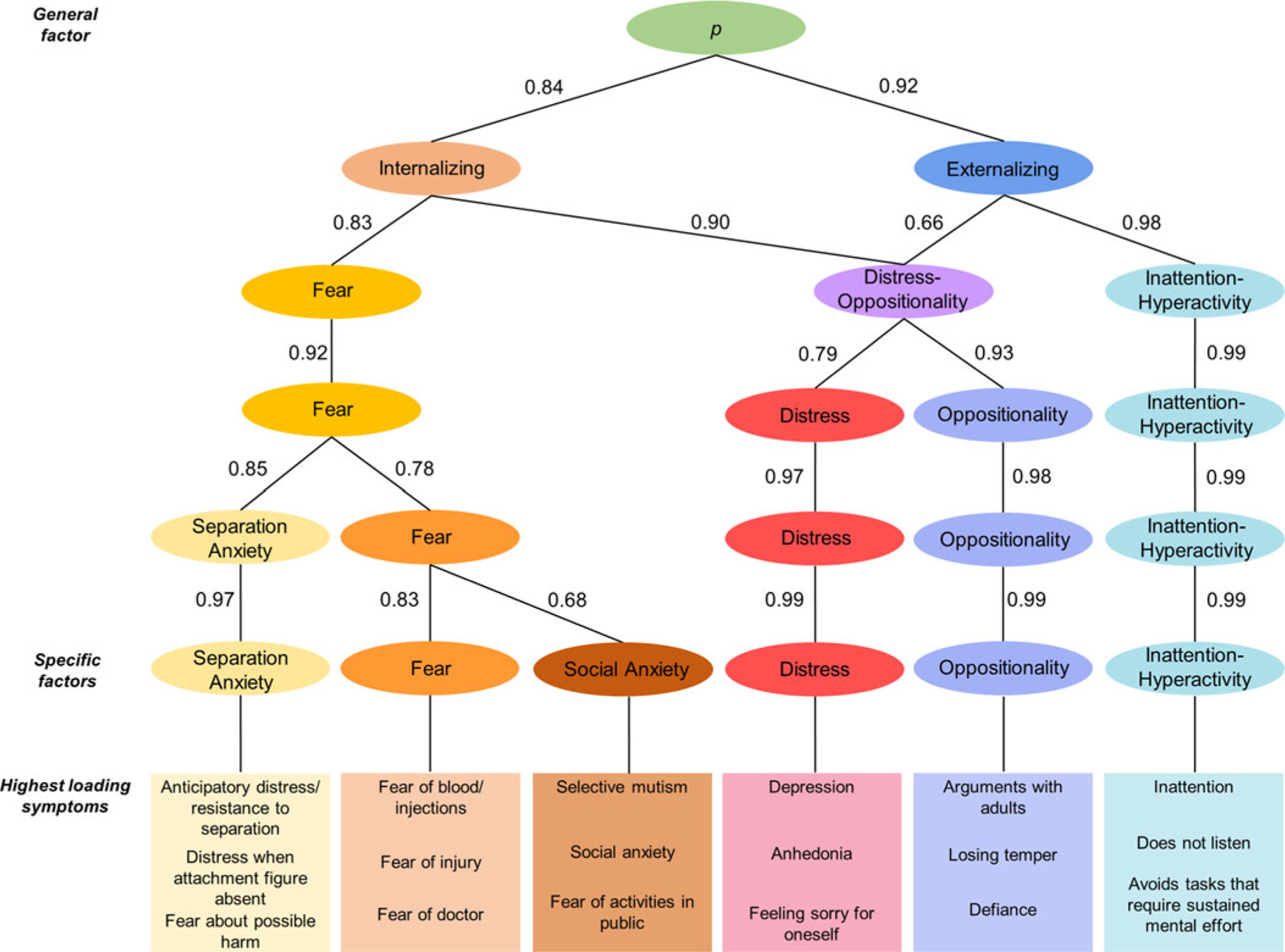
Schematic representation of the hierarchical structure of preschool psychopathology derived by extracting and correlating factor solutions with an increasing number of factors. *Note*: Arrows depict correlations >0.60 with shift across hierarchical levels of more than 2 primary-loading items, indicating a shift in content from a higher-level factor to a lower-level factor. Analyses were run combining all three samples.

A total of 76 symptoms were included in the EFA. After examining the results of parallel analyses (Figure S1) and the interpretability of factor solutions, models including 1 to 6 factors were found acceptable (Fig. 1, online Supplementary Table S1), as more complex models included at least one factor with less than 4 primary loadings and showed poor interpretability.

Models from 1-factor to 6-factors were arranged in a hierarchical structure, with paths showing substantial correlations between levels (Fig. 1). All correlations within and across hierarchical levels are given in online Supplementary Table S2. The 1-factor structure reflected a general preschool *p* factor, with strong loadings on most PAPA symptoms (online Supplementary Table S1). The 2-factor solution showed broad internalizing and externalizing factors. In the 3-factor structure, a distress-oppositionality factor (e.g. irritability, tantrums, depression) emerged from the broad internalizing and externalizing factors, alongside fear and inattention-hyperactivity factors. In the 4-factor solution, the distress-oppositionality factor split into distress (e.g. depression, tearfulness) and oppositionality (e.g. tantrums, arguments with adults) factors. In the 5-factor structure, separation anxiety (e.g. distress in absence of caregiver) split from other fear problems (e.g. fear of blood/injections, social anxiety). Finally, in the 6-factor structure, the social anxiety split from fear. This most differentiated interpretable factor solution was used to measure specific preschool dimensions in subsequent prospective analyses.

The additional confirmatory bifactor model yielded general and specific factors that were highly correlated with the factors from EFA (*p* factors: *r* = 0.98; specific factors: *r* = 0.62–0.81, all *p* < 0.01; online Supplementary Material, Table S3). Thus, only factors from EFA were used in subsequent analyses.

### Preschool dimensions and preadolescent outcomes

#### Psychiatric disorders

**Fig. 2.**
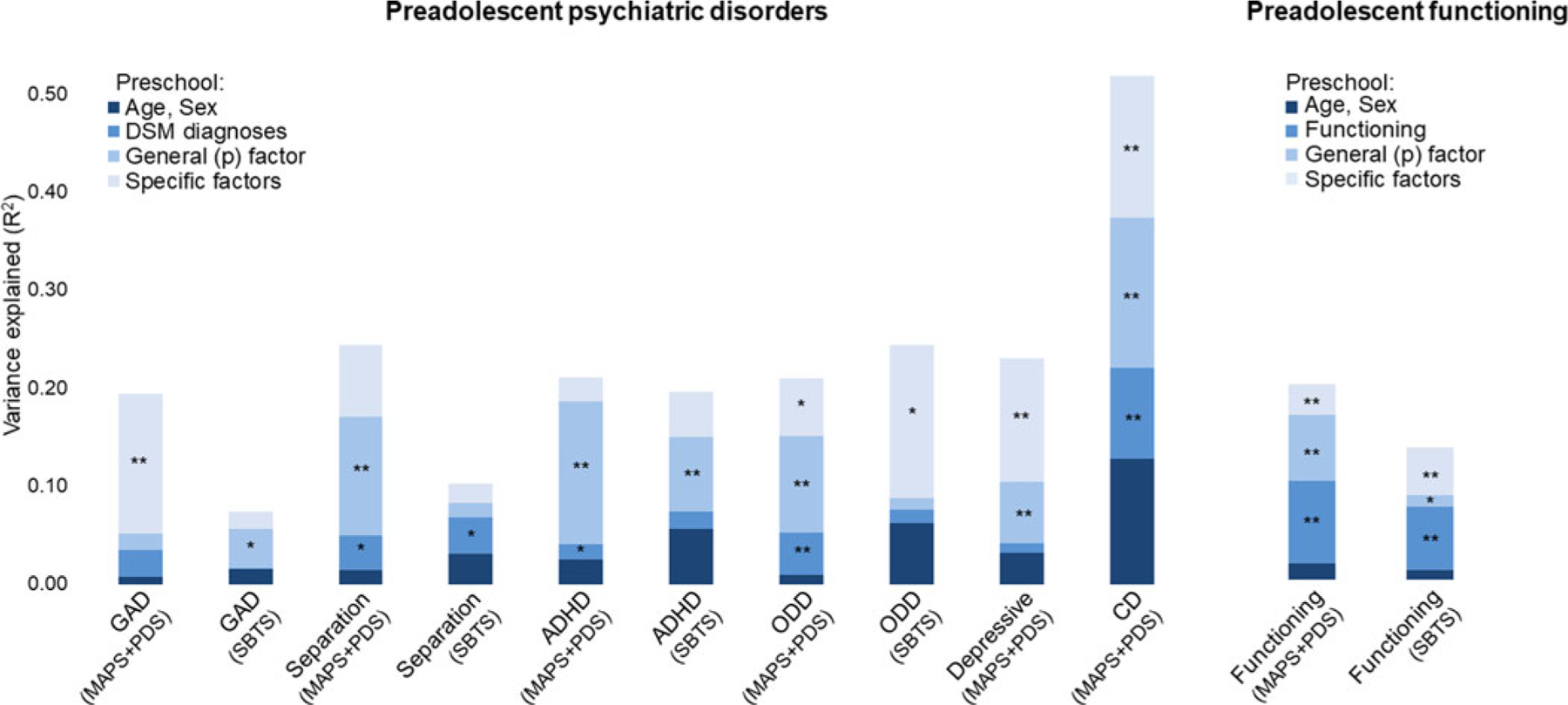
Variance in preadolescence disorders explained by preschool DSM diagnoses, general (p) factor, and specific factors of psychopathology. *Notes*: Nagelkerke pseudo-*R*^2^ is plotted for preadolescent psychiatric disorders. Asterisks (**p < 0.01, *p < 0.05) indicate that adding a block yielded a statistically significant change in *R*^2^ from the previous block in hierarchical regression models. Depressive disorders and CD were investigated only in MAPS + PDS as too few SBTS participants met the criteria for these conditions in preadolescence. ADHD, attention-deficit/hyperactivity disorder; CD, conduct disorder; GAD, generalized anxiety disorder; MAPS, Multidimensional Assessment of Preschoolers Study; ODD, oppositional defiant disorder; PDS, Preschool Depression Study; SBTS, Stony Brook Temperament Study.

Preadolescent psychiatric outcomes were examined with hierarchical logistic regressions. Preschool psychiatric diagnoses were generally weak and inconsistent predictors of preadolescent disorders in both MAPS + PDS and SBTS (Fig. 2; see also online Supplementary Tables S4 and S5 for analyses separating MAPS and PDS). The preschool *p* factor (1-factor model) prospectively predicted all preadolescent psychiatric diagnoses over and above preschool diagnoses in either MAPS + PDS or SBTS, with significant increases in *R*^2^ ranging between 4% and 15% after adding the *p* factor (Fig. 2, online Supplementary Table S4). The exceptions were GAD in MAPS + PDS and separation anxiety disorder and ODD in SBTS, where adding the *p* factor did not significantly increase the *R*^2^.

When adding factors solutions with 2 to 6 factors, we broadly found that the greater the number of factors, the greater the variance explained (online Supplementary Table S6). The 6 specific factors accounted for a significantly larger proportion of variance in preadolescent disorders over the *p* factor and DSM diagnoses, with *R*^2^ increasing 6–16% after adding specific factors (Fig. 2, online Supplementary Table S4). The only preadolescent disorders not showing a significant improvement in *R*^2^ when adding the 6-factor model were separation anxiety disorder and ADHD across samples, and GAD in SBTS.

Results in SBTS excluding participants with preschool diagnoses were largely consistent (online Supplementary Material, Table S4).

In models that examined each factor individually, higher scores on the preschool *p* factor were prospectively associated with all preadolescent disorders [odds ratios (ORs) = 1.87–6.04, *p* < 0.05], except GAD and ODD in SBTS (Table 2; online Supplementary Table S7 for analyses separating MAPS and PDS). Specific preschool dimensions showed few significant effects in SBTS, but numerous effects in MAPS + PDS (Tables 2, online Supplementary Table S7). Higher preschool distress, separation anxiety, inattention-hyperactivity, and oppositionality factors were predictive of most future diagnoses, whereas the fear and social anxiety factors showed more specific associations (Table 2).

#### Functioning

Functional outcomes were examined in hierarchical linear regressions. The preschool *p* factor prospectively predicted preadolescent functional impairment over and above preschool functioning (significant *R*^2^ increase of 7% in MAPS + PDS and of 1% in SBTS; Figure 2, online Supplementary Tables S4–S5). Adding the 6-factor solution produced a further significant *R*^2^ increase (4% in MAPS + PDS and 5% in SBTS). *R*^2^ also increased, albeit not always significantly, when a greater number of factors were included beyond the *p* factor (online Supplementary Table S6). As a control analysis, we ran hierarchical regressions to test whether preschool DSM diagnoses (entered as block 3) predicted preadolescent functional impairments controlling for preschool age and sex (block 1) and preschool functioning (block 2). Preschool diagnoses were significant predictors beyond preschool functioning in SBTS (*R*^2^ increase of 1%), but not in MAPS + PDS (online Supplementary Tables S4–S5). However, these models including DSM diagnoses explained a significantly smaller proportion of variance in preadolescence functioning than models including general and specific factors (MAPS +PDS: Hotelling’s *t* = 4.24, *p* < 0.001; SBTS: Hotelling’s *t* = 2.29, *p* = .02).

In analyses examining each factor individually, higher scores on the *p* factor and all specific dimensions prospectively predicted worse functioning in preadolescence (standardized *β* = 0.14–0.38, *p* < 0.05), except for social anxiety in MAPS + PDS and fear in both MAPS + PDS and SBTS (Tables 2, online Supplementary Table S5).

## Discussion

The current study represents the most comprehensive examination to date of the structure and predictive validity of transdiagnostic hierarchical dimensions of psychopathology in early childhood. We identified a dimensional hierarchy with the *p* factor at the apex and six lower-level specific factors, capturing distress, fear, social anxiety, separation anxiety, inattention-hyperactivity, and oppositionality. Higher levels of the *p* factor and specific factors prospectively predicted new onsets of psychiatric disorders as well as functional impairment in preadolescence – almost a decade later – over and above preschool DSM diagnoses and functioning. These findings highlight the value of conceptualizing preschool psychopathology using data-driven hierarchical models of dimensional phenotypes that capture individual differences in severity for forecasting important clinical and functional outcomes. The identified preschool dimensions of psychopathology may be used to guide future early identification and prevention strategies to reduce the long-lasting impact of preschool psychiatric symptoms.

Our study delineated a multi-level transdiagnostic hierarchical structure of psychopathology, with a *p* factor at the top (Caspi et al., 2014; Lahey et al., 2012; Michelini et al., 2019), which separated into the broad externalizing and internalizing spectra in the 2-factor structure (Achenbach et al., 2017). The broad externalizing factor progressively split into oppositionality and inattention-hyperactivity factors, whereas the broad internalizing factors split into specific distress, fear, separation anxiety, and social anxiety factors. These findings extend the few prior studies investigating both general and specific dimensions in early childhood, where fewer specific factors were specified a-priori, using a bifactor approach (McElroy et al., 2018; Olino et al., 2014). Most of the specific dimensions identified by our data-driven approach captured symptoms belonging to specific diagnostic categories, whereas the distress factor aggregated symptoms of both depressive disorders and GAD. These findings in preschoolers are broadly consistent with previous studies of older children and adults, which converged in the HiTOP model (Kotov et al., 2017), and support the use of transdiagnostic hierarchical approaches across development (Forbes et al., 2019; Wakschlag et al., 2019; Wilson & Olino, 2021).

Using preadolescent follow-up assessments, we compared the ability of the preschool *p* factor and specific psychopathology dimensions to predict future clinical and functional outcomes. We found weak and inconsistent evidence that preschool DSM diagnoses predicted preadolescent disorders, as indicated by the small portion of variance explained beyond covariates. Conversely, the preschool *p* factor predicted preadolescent disorders over and above preschool diagnoses, with only a few exceptions for GAD in MAPS + PDS and separation anxiety disorder and ODD in SBTS, suggesting that general preschool psychopathology severity may have limited predictive power in these outcomes beyond the presence of a preschool diagnosis. Further, specific dimensions significantly predicted preadolescent disorders beyond p, except for preadolescent separation anxiety disorder and ADHD. A similar pattern of findings emerged in predicting preadolescent functional impairment, where both the preschool *p* factor and specific dimensions were significant predictors even when controlling for preschool functioning and to a greater extent than preschool DSM diagnoses. These results in early childhood are consistent with initial findings on the predictive validity of the *p* factor at school age (Pettersson et al., 2018) and with models conceptualizing the *p* factor as a common liability to all form of psychopathology (Caspi et al., 2014; Lahey, Krueger, Rathouz, Waldman, & Zald, 2017).

Our novel findings have two important clinical implications. First, assessment of both general and specific dimensions of preschool psychopathology offers greater prognostic utility than binary DSM diagnoses, supporting a shift away from the exclusive use of diagnostic categories for predicting long-term outcomes. This is because these broad dimensions reflect the continuous nature of psychopathology and can accommodate subthreshold presentations. Second, early interventions and prevention strategies targeting preschool general psychopathology may be able to avert the emergence of various psychiatric disorders over the next decade, but targeting specific dimensions may improve certain outcomes further. Future intervention studies should test whether a transdiagnostic stepped-care approach for preschool children, firstly aiming to reduce general psychopathology and secondly targeting more specific problems, may represent an effective strategy for preventing subsequent psychiatric conditions throughout development (Forbes et al., 2019; Wakschlag et al., 2019).

The present study has the following limitations. First, the preschool dimensions of psychopathology were derived from diagnostic assessments based on one informant (usually mothers). Although this limitation is common to much of the existing literature in early childhood, future research should confirm these findings using additional informants, such as fathers and teachers, or observational assessments. Second, our study aggregated data from three samples recruited with different methods and assessed with partly overlapping measures. Different findings for some preadolescent disorders in SBTS (community sample, predominantly middle-class and White) *v*. MAPS + PDS (psychopathology-enriched samples, socio-demographically and racially diverse) may be explained by differences in sample composition. Nevertheless, our multi-sample approach yielded a large sample size, necessary for factor analysis of a large number of symptoms, and provided convergent findings across different methodologies and recruitment strategies. Finally, our study only focused on one follow-up point in preadolescence, which was available in all three studies. Future studies examining multiple follow-up points will be helpful for clarifying how the predictive power of preschool dimensions changes for outcomes in different developmental periods.

## Conclusion

Our study provides novel findings on the hierarchical dimensional structure of preschool psychopathology in a large and diverse sample of young children. The identified *p* and specific dimensions show substantial predictive validity with regard to important clinical and functional outcomes in preadolescence, up to almost a decade later. Of note, these dimensions were substantially more predictive of outcomes than DSM diagnoses, although both dimensions and diagnoses were derived from the same preschool interview. An important next step will be to translate these findings into clinical practice, for example through the development of clinical cutoffs and predictive algorithms in independent samples. Future efforts should also test whether targeting the identified hierarchical dimensions through preventive interventions may improve the developmental trajectories of preschool children at risk for persistent psychopathology.

## Supplementary Material

Supplementary Materials

## Figures and Tables

**Table 1.** Demographic characteristics and rates of psychiatric diagnoses in each sample

	MAPS	PDS	SBTS
*N* at baseline	410	302	541
*N* at follow-up	298	235	434
Sex: *N* female (% female)	207 (50.5%)	145 (48%)	248 (45.8%)
Age at baseline: mean (s.d.)	4.65 (0.85)	4.45 (0.80)	3.62 (0.27)
Age at follow-up: mean (s.d.)	9.20 (0.85)	11.17 (0.87)	12.80 (0.45)
Any DSM diagnosis at baseline: *N* (%)	321 (78.3%)	153 (50.1%)	148 (27.4%)
DSM diagnosis at follow-up: *N* (%)	51 (17.1%)	69 (29.4%)	73 (16.8%)
GAD	5 (1.7%)	16 (6.8%)	20 (4.6%)
Separation anxiety disorder	11 (3.7%)	6 (2.6%)	16 (3.7%)
Depressive disorder	2 (0.7%)	44 (18.7%)	5 (1.2%)
ADHD	26 (8.7%)	34 (14.5%)	42 (9.7%)
ODD	28 (9.4%)	23 (9.8%)	13 (3%)
CD	4 (1.3%)	8 (3.4%)	0 (0%)

ADHD, attention-deficit/hyperactivity disorder; CD, conduct disorder; GAD, generalized anxiety disorder; MAPS, Multidimensional Assessment of Preschoolers Study; N, number of participants; ODD, oppositional defiant disorder; PDS, Preschool Depression Study; SBTS, Stony Brook Temperament Study; s.d., standard deviation.

*Notes*: current preadolescent diagnoses were used in analyses of MAPS and PDS. Diagnoses met in the interval between assessments carried out at age 9 and at age 12 (i.e. interval diagnoses) were instead analyzed in SBTS, due to the lower rates of preadolescent diagnoses in this population-based sample. As only a subset of psychiatric diagnoses was measured at follow-up, the rates of diagnoses at follow-up do not reflect the total number of participants who met the criteria for any DSM diagnosis in preadolescence.

**Table 2.** Bivariate associations of preschool psychopathology dimensions (rows) with preadolescent psychiatric and functional outcomes (columns)

	GAD	Separation anxiety disorder	ADHD	ODD	Depressive disorder	CD	Functional impairment
	OR	OR	OR	OR	OR	OR	*β*
General (p) factor							
MAPS+PDS	1.87*	4.03**	3.09**	3.07**	2.16**	6.04**	0.38**
SBTS	1.67	2.08*	2.55**	1.74			0.24**
Distress							
MAPS+PDS	3.03**	2.33**	1.90**	1.58**	2.46**	4.25**	0.31**
SBTS	1.54	1.44	1.46	1.45			0.20**
Separation anxiety							
MAPS+PDS	1.42*	2.04**	1. 35*	1.67**	1.51**	2.39**	0.14*
SBTS	1.03	1.34	1.46*	1.80*			0.20**
Social anxiety							
MAPS+PDS	1.42	2.45**	1.15	1.09	1.06	1.08	0.08
SBTS	0.98	0.99	1.03	1.07			0.22**
Fear							
MAPS+PDS	0.78	1.73*	0.94	1.33	0.58**	1.26	0.01
SBTS	0.98	2.10*	1.54	0.41			0.01
Inattention-hyperactivity							
MAPS+PDS	1.06	2.30**	2.38**	2.00**	1.40*	2.37*	0.29**
SBTS	1.67	1.83*	2.80**	1.18			0.22**
Oppositionality							
MAPS+PDS	1.53	2.58**	2.31**	3.22**	2.12**	4.92**	0.37**
SBTS	1.31	1.71*	1.43*	2.12*			0.16**

ADHD, attention-deficit/hyperactivity disorder; CD, conduct disorder; GAD, generalized anxiety disorder; MAPS, Multidimensional Assessment of Preschoolers Study; ODD, oppositional defiant disorder; OR, odds ratio; PDS, Preschool Depression Study; SBTS, Stony Brook Temperament Study.

*Notes*:

* *p* < 0.05,

** *p* < 0.01.

All analyses were run on standardized variables and controlled for sex and preschool age. CGAF functioning scores were reverse-coded, such that higher scores indicate worse functioning, similar to the HBQ scores in MAPS + PDS. Depressive disorders and CD were investigated only in MAPS + PDS as too few SBTS participants met the criteria for these conditions.
