# Supplementary material for: Do general and specific factors of preschool psychopathology predict preadolescent outcomes? A transdiagnostic hierarchical approach
Source: Psychol Med. Author manuscript; Available in PMC 2023 Oct 6. (PMC10482704; doi:10.1017/S003329172200246X)
Supplement: Supplementary Materials [file NIHMS1887860-supplement-Supplementary_Materials.docx]

**Supplementary Material**

*“Do general and specific factors of preschool psychopathology predict preadolescent outcomes? A transdiagnostic hierarchical approach.”*

**Data harmonization**

The Preschool Age Psychiatric Assessment by trained interviewers (PAPA) (Egger et al., 2006) was used with small modifications in the three studies. The full PAPA was administered in the Multidimensional Assessment of Preschoolers Study (MAPS) and the Preschool Depression Study (PDS), including sections assessing generalized anxiety disorder (GAD), separation anxiety disorder, specific phobias, agoraphobia, social phobia, depressive disorders, post-traumatic stress disorder, panic disorder, conduct disorder (CD), oppositional defiant disorder (ODD), attention deficit hyperactivity disorder (ADHD). Preschool assessments with the PAPA in Stony Brook Temperament Study (SBTS) did not include the CD section. Some items were also skipped across all studies. As such, we first compared all items included across the three samples and identified a common set of symptoms assessed in all participants. In the first half of the SBTS sample, the ADHD and ODD sections of the PAPA were not administered in full to participating parents who did not endorse at least one symptom of either disorder in a screening questionnaire, the Child Symptom Inventory (Gadow & Sprafkin, 1997). For participants whose parent did not complete the full ADHD and ODD sections, we imputed data for PAPA ADHD and ODD symptoms using expectation-maximization (EM) algorithm and used these data in subsequent analyses.

**Data preparation**

To address problems in EFA with items that were not analyzable due being endorsed too infrequently or being too-highly correlated with other items (Floyd & Widaman, 1995), we took two approaches. Firstly, we examined frequencies of all symptoms included in the PAPA and removed items for which frequency was too low (>98.5% rated 0). Secondly, to address high inter-item correlations, which can distort factor structure, we aggregated items that were highly correlated (polychoric r>.75) into composites by creating maximum scores (i.e., the highest score among correlated items).

The following PAPA items were removed because of low frequency: “Delusions of guilt”, “Free floating anxious affect”, “Motor slowing”, “Hopelessness”, “Suicide themes in play”, “Fear about calamitous separation”, “Physical symptoms of separation”, and “Lack of protest”. We also created the following composites: “Inattention” (composite of “Difficulty concentrating on tasks”, “Difficulty concentrating on adult directed tasks”, “Easily distracted by extraneous stimuli”), “Tiredness” (composite of “Tiredness”, “Fatiguability”, “Anergia”), “Anhedonia” (composite of “Loss of interest”, “Anhedonia”), “Depressed” (composite of “Depressed mood”, “Looks unhappy”).

**Confirmatory bifactor model and comparison of factors from the exploratory models**

We ran an additional confirmatory bifactor model analysis of the PAPA items in Mplus using the weighted least squares means and variance adjusted (WLSMV) algorithm. This model included a general p factor and 6 specific factors, following the results of the ESEM analyses. Each item received loadings from the general factor and also from the factor that showed the highest item loading in the 6-factor model in exploratory analyses, shown in Table S1. For example, in the bifactor model, the item “Depressed (composite)” had loadings from the general factor and also the specific Distress factor. Model fit information for this model was: free parameters=305; χ^2^=3984.71, p<.01; Root Mean Square Error of Approximation (RMSEA)=.02, 90% confidence interval=.018-.021; Comparative Fit Index (CFI)=.95; Tucker-Lewis index (TLI)=.94.

The p factors from the bifactor and exploratory models were virtually identical, with correlation approaching unity (r=.98, p<.01). Similarly, the specific factors from the bifactor and confirmatory models were highly correlated (range between r=.62-.81, all p<.01). Correlations were even higher when regressing out the p factor (from the exploratory model) from specific factors, which approximates the orthogonal nature of general and specific factors in bifactor models, and correlating these residuals with the specific factors from the bifactor model (range between r=.72-.91, all p<.01). All correlations between specific factors are reported in Table S3.

**Prediction of first-onset psychiatric disorders in SBTS**

In an additional analysis in SBTS participants, we reran hierarchical regression models after excluding excluded participants meeting criteria for any diagnosis at baseline to investigate the effects of preschool dimensions on first onset of psychiatric disorders. We did not use MAPS and PDS in this analysis, as excluding participants with preschool diagnoses would have resulted in too small an N in these samples enriched for preschool psychopathology (Table 1).

Among SBTS participants with no DSM diagnoses at baseline (N=393), N=15 participants had a pre-adolescent GAD diagnosis, N=8 had a pre-adolescent separation anxiety disorder diagnosis, N=24 had a pre-adolescent ADHD diagnosis, and N=7 had a pre-adolescent ODD diagnosis. The preschool p factor alone significantly predicted first onset of ADHD, whereas the addition of specific factors did not improve variance explained (Table S3). Neither preschool DSM nor p or specific factors prospectively predicted first onsets of other disorders in preadolescence (Table S3). Area under the curve (AUC) values and chance in AUC between blocks are reported for completeness but should be interpreted cautiously given the low rates of preadolescent first onsets, especially of separation anxiety disorder and ODD.

**Figure S1. Polychoric parallel analysis of PAPA symptoms, showing that up to 7 factors can be extracted.**


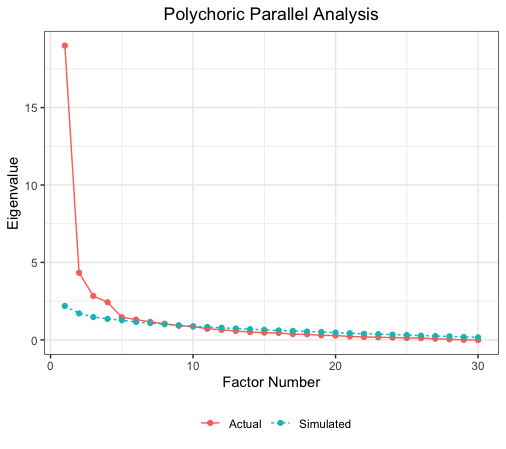


Note: the simulated line represents the top 95% confidence interval around simulated eigenvalues.

**Table S1. Factor loadings for the factor solutions from exploratory factor analyses of preschool symptoms.**

|  | **1-factor** | **2-factor** | | **3-factor** | | | **4-factor** | | | | **5-factor** | | | | | **6-factor** | | | | | |
| --- | --- | --- | --- | --- | --- | --- | --- | --- | --- | --- | --- | --- | --- | --- | --- | --- | --- | --- | --- | --- | --- |
|  | F1 | F1 | F2 | F1 | F2 | F3 | F1 | F2 | F3 | F4 | F1 | F2 | F3 | F4 | F5 | F1 | F2 | F3 | F4 | F5 | F6 |
|  | p | Int | Ext | Fear | Distr-Opp | Inatt-Hyp | Distr | Fear | Inatt-Hyp | Opp | Sep Anx | Distr | Fear | Inatt-Hyp | Opp | Distr | Sep Anx | Soc Anx | Inatt-Hyp | Fear | Opp |
| **Factor loadings** | | | | | | | | | | | | | | | | | | | | | |
| Depressed (composite) | **0.64** | **0.77** | -0.01 | 0.30 | **0.67** | -0.14 | **0.77** | 0.00 | -0.11 | 0.22 | 0.11 | **0.78** | -0.10 | -0.06 | 0.17 | **0.76** | 0.06 | 0.06 | 0.02 | -0.20 | 0.15 |
| Anhedonia (composite) | **0.59** | **0.63** | 0.08 | 0.29 | **0.52** | -0.02 | **0.72** | -0.05 | 0.04 | 0.08 | 0.02 | **0.72** | -0.05 | 0.06 | 0.06 | **0.70** | -0.03 | 0.09 | 0.16 | -0.17 | 0.03 |
| Feeling sorry for oneself | **0.53** | **0.50** | 0.14 | 0.18 | **0.49** | 0.03 | **0.68** | -0.17 | 0.08 | 0.06 | -0.14 | **0.68** | -0.04 | 0.08 | 0.08 | **0.68** | -0.10 | -0.13 | 0.06 | 0.02 | 0.09 |
| Self- depreciation | **0.53** | **0.54** | 0.10 | 0.22 | **0.50** | -0.01 | **0.67** | -0.07 | 0.03 | 0.08 | -0.09 | **0.66** | 0.02 | 0.02 | 0.10 | **0.65** | -0.10 | 0.00 | 0.04 | 0.00 | 0.11 |
| Tiredness (composite) | **0.43** | **0.37** | 0.15 | 0.26 | 0.25 | 0.09 | **0.65** | -0.15 | 0.17 | -0.16 | -0.16 | **0.65** | 0.04 | 0.15 | -0.14 | **0.64** | -0.19 | 0.03 | 0.21 | -0.01 | -0.14 |
| Pathological guilt | **0.44** | **0.54** | -0.02 | 0.27 | **0.43** | -0.10 | **0.58** | 0.02 | -0.07 | 0.08 | 0.12 | **0.63** | -0.10 | 0.00 | 0.00 | **0.64** | 0.15 | -0.10 | -0.04 | -0.05 | 0.01 |
| Reported tearfulness and crying | **0.58** | **0.75** | -0.07 | 0.25 | **0.70** | -0.20 | **0.66** | 0.04 | -0.20 | 0.32 | 0.11 | **0.62** | -0.03 | -0.18 | 0.32 | **0.61** | 0.07 | 0.06 | -0.15 | -0.10 | 0.32 |
| Separation dreams | 0.31 | **0.50** | -0.13 | 0.32 | 0.30 | -0.18 | **0.56** | 0.06 | -0.13 | -0.05 | 0.06 | **0.58** | 0.04 | -0.10 | -0.07 | **0.61** | 0.10 | -0.10 | -0.19 | 0.13 | -0.03 |
| Feels unloved | **0.57** | **0.48** | 0.20 | 0.28 | 0.37 | 0.12 | **0.47** | 0.08 | 0.15 | 0.11 | 0.12 | **0.49** | 0.00 | 0.19 | 0.05 | **0.51** | 0.16 | -0.09 | 0.14 | 0.06 | 0.06 |
| Boredom | **0.53** | **0.42** | 0.21 | 0.27 | 0.31 | 0.14 | **0.50** | 0.03 | 0.18 | 0.03 | 0.00 | **0.49** | 0.08 | 0.17 | 0.03 | **0.49** | 0.00 | 0.02 | 0.18 | 0.06 | 0.04 |
| Headaches | **0.43** | **0.35** | 0.16 | 0.27 | 0.21 | 0.12 | **0.48** | 0.02 | 0.16 | -0.08 | -0.07 | **0.46** | 0.16 | 0.12 | -0.04 | **0.47** | -0.05 | -0.03 | 0.07 | 0.21 | 0.00 |
| Worries | **0.58** | **0.55** | 0.15 | 0.37 | 0.36 | 0.08 | **0.49** | 0.16 | 0.11 | 0.10 | 0.07 | **0.44** | 0.20 | 0.07 | 0.14 | **0.45** | 0.06 | 0.09 | 0.04 | 0.17 | 0.16 |
| Daytime sleepiness | **0.35** | 0.27 | 0.15 | 0.19 | 0.19 | 0.10 | **0.39** | -0.03 | 0.14 | -0.04 | -0.08 | **0.38** | 0.09 | 0.11 | -0.02 | **0.39** | -0.05 | -0.05 | 0.08 | 0.12 | 0.00 |
| Loneliness | **0.49** | **0.42** | 0.17 | **0.39** | 0.17 | 0.14 | **0.43** | 0.16 | 0.20 | -0.07 | 0.12 | **0.44** | 0.13 | 0.21 | -0.11 | **0.45** | 0.11 | 0.06 | 0.19 | 0.11 | -0.10 |
| Nervous affect | **0.55** | **0.61** | 0.05 | **0.45** | 0.32 | 0.01 | 0.41 | 0.31 | 0.03 | 0.12 | 0.21 | 0.34 | 0.25 | 0.00 | 0.14 | **0.35** | 0.15 | 0.22 | 0.01 | 0.14 | 0.15 |
| Indecisiveness | **0.50** | **0.43** | 0.16 | 0.33 | 0.25 | 0.12 | **0.37** | 0.17 | 0.14 | 0.06 | 0.13 | **0.35** | 0.13 | 0.14 | 0.05 | **0.35** | 0.09 | 0.14 | 0.17 | 0.04 | 0.04 |
| Anticipatory distress resistance to separation | **0.41** | **0.63** | -0.13 | **0.55** | 0.18 | -0.11 | -0.11 | **0.67** | -0.13 | 0.37 | **0.80** | 0.00 | -0.04 | 0.03 | 0.09 | 0.02 | **0.78** | 0.11 | -0.05 | -0.05 | 0.09 |
| Distress when absent figure absent | **0.46** | **0.55** | 0.01 | **0.62** | 0.00 | 0.07 | -0.24 | **0.71** | 0.10 | 0.26 | **0.75** | -0.11 | 0.05 | 0.25 | -0.02 | -0.07 | **0.76** | 0.04 | 0.11 | 0.12 | -0.01 |
| Fear about Possible Harm | **0.48** | **0.59** | -0.01 | **0.55** | 0.15 | 0.01 | 0.00 | **0.58** | 0.01 | 0.27 | **0.71** | 0.16 | -0.10 | 0.19 | -0.04 | 0.19 | **0.74** | -0.05 | 0.06 | -0.01 | -0.03 |
| Social withdrawal when attachment figure absent | **0.48** | **0.59** | -0.01 | **0.57** | 0.11 | 0.03 | -0.08 | **0.62** | 0.03 | 0.28 | **0.68** | 0.02 | 0.03 | 0.16 | 0.04 | 0.03 | **0.66** | 0.12 | 0.11 | 0.00 | 0.03 |
| Avoidance of being alone | **0.57** | **0.47** | 0.21 | **0.50** | 0.11 | 0.21 | 0.13 | **0.45** | 0.23 | 0.11 | **0.41** | 0.15 | 0.18 | 0.27 | 0.00 | 0.18 | **0.42** | 0.07 | 0.18 | 0.21 | 0.02 |
| Selective mutism | 0.27 | **0.60** | -0.26 | **0.64** | -0.02 | -0.17 | 0.16 | **0.59** | -0.12 | -0.06 | 0.34 | -0.03 | **0.52** | -0.21 | 0.07 | -0.05 | 0.09 | **0.74** | 0.03 | -0.01 | 0.01 |
| Social anxiety | **0.36** | **0.60** | -0.16 | **0.67** | -0.04 | -0.07 | 0.21 | **0.59** | -0.01 | -0.11 | 0.26 | 0.01 | **0.61** | -0.13 | 0.05 | 0.02 | 0.06 | **0.67** | 0.02 | 0.21 | 0.03 |
| Fear of activities in public | **0.40** | **0.66** | -0.17 | **0.72** | 0.00 | -0.09 | 0.32 | **0.58** | -0.02 | -0.14 | 0.24 | 0.13 | **0.62** | -0.15 | 0.02 | 0.15 | 0.05 | **0.62** | -0.03 | 0.25 | 0.01 |
| Agoraphobia | **0.38** | **0.58** | -0.12 | **0.53** | 0.14 | -0.10 | 0.19 | **0.50** | -0.08 | 0.09 | 0.29 | 0.03 | **0.44** | -0.16 | 0.20 | 0.02 | 0.12 | **0.54** | -0.04 | 0.12 | 0.19 |
| Inattention (composite) | **0.79** | 0.01 | **0.84** | 0.17 | -0.01 | **0.81** | 0.04 | 0.07 | **0.82** | 0.04 | 0.06 | 0.06 | 0.09 | **0.82** | -0.01 | 0.03 | 0.05 | 0.07 | **0.87** | 0.01 | -0.06 |
| Does not listen | **0.73** | -0.01 | **0.80** | -0.07 | 0.20 | **0.72** | 0.05 | -0.14 | **0.70** | 0.23 | 0.01 | 0.10 | -0.17 | **0.73** | 0.15 | 0.05 | -0.01 | 0.01 | **0.85** | -0.29 | 0.08 |
| Avoid tasks that require sustained mental effort | **0.59** | -0.07 | **0.69** | 0.15 | -0.12 | **0.68** | 0.02 | 0.04 | **0.71** | -0.07 | -0.01 | 0.02 | 0.12 | **0.69** | -0.09 | -0.02 | -0.06 | 0.17 | **0.79** | -0.03 | -0.14 |
| Fails to pay close attention to details | **0.71** | 0.01 | **0.75** | 0.17 | -0.01 | **0.72** | 0.13 | 0.01 | **0.74** | -0.03 | -0.03 | 0.14 | 0.10 | **0.72** | -0.05 | 0.12 | -0.04 | 0.06 | **0.78** | 0.03 | -0.09 |
| difficulty remaining seated | **0.75** | -0.01 | **0.82** | -0.03 | 0.16 | **0.75** | -0.07 | -0.03 | **0.72** | 0.27 | 0.07 | -0.02 | -0.10 | **0.75** | 0.19 | -0.04 | 0.12 | -0.11 | **0.76** | -0.08 | 0.14 |
| Behavioral blurting | **0.63** | -0.06 | **0.74** | -0.13 | 0.19 | **0.67** | -0.09 | -0.10 | **0.63** | 0.30 | 0.06 | -0.03 | -0.20 | **0.68** | 0.20 | -0.06 | 0.09 | -0.11 | **0.73** | -0.21 | 0.15 |
| Difficulty following instructions | **0.75** | 0.11 | **0.72** | 0.12 | 0.15 | **0.66** | 0.15 | -0.01 | **0.66** | 0.13 | -0.02 | 0.13 | 0.07 | **0.64** | 0.12 | 0.10 | -0.05 | 0.11 | **0.72** | -0.04 | 0.09 |
| Difficulty organizing tasks | **0.74** | 0.13 | **0.70** | 0.23 | 0.06 | **0.65** | 0.15 | 0.09 | **0.67** | 0.04 | 0.02 | 0.12 | 0.18 | **0.64** | 0.04 | 0.10 | -0.01 | 0.14 | **0.70** | 0.07 | 0.01 |
| Difficulty waiting for turn | **0.74** | 0.12 | **0.71** | 0.03 | 0.25 | **0.63** | -0.04 | 0.05 | **0.60** | 0.34 | 0.13 | -0.03 | -0.04 | **0.61** | 0.29 | -0.07 | 0.11 | 0.06 | **0.69** | -0.14 | 0.24 |
| Difficulty doing things quietly | **0.72** | -0.04 | **0.81** | 0.03 | 0.08 | **0.75** | -0.07 | 0.01 | **0.74** | 0.19 | 0.11 | -0.01 | -0.08 | **0.77** | 0.08 | 0.02 | 0.24 | -0.30 | **0.68** | 0.10 | 0.05 |
| Often blurts out answers to questions | **0.58** | -0.05 | **0.68** | -0.02 | 0.08 | **0.63** | 0.07 | -0.12 | **0.63** | 0.08 | -0.06 | 0.11 | -0.07 | **0.64** | 0.03 | 0.12 | 0.03 | -0.23 | **0.61** | 0.04 | 0.00 |
| Often interrupts | **0.68** | 0.05 | **0.70** | 0.00 | 0.19 | **0.63** | -0.03 | 0.00 | **0.60** | 0.27 | 0.06 | -0.01 | -0.04 | **0.61** | 0.22 | -0.01 | 0.11 | -0.13 | **0.59** | 0.02 | 0.19 |
| Forgetful in daily activities | **0.64** | 0.17 | **0.56** | 0.22 | 0.09 | **0.52** | 0.29 | 0.01 | **0.56** | -0.04 | -0.12 | 0.24 | 0.21 | **0.49** | 0.02 | 0.22 | -0.17 | 0.17 | **0.58** | 0.08 | -0.01 |
| Accident prone | **0.53** | 0.01 | **0.57** | 0.07 | 0.05 | **0.53** | 0.11 | -0.05 | **0.55** | 0.02 | -0.06 | 0.11 | 0.05 | **0.53** | 0.02 | 0.11 | -0.04 | -0.03 | **0.55** | 0.05 | -0.01 |
| Talks excessively | **0.57** | -0.03 | **0.65** | 0.06 | 0.02 | **0.62** | -0.05 | 0.03 | **0.61** | 0.10 | 0.09 | 0.00 | -0.03 | **0.64** | 0.02 | 0.04 | 0.21 | -0.27 | **0.52** | 0.15 | 0.01 |
| Often loses things | **0.56** | 0.19 | **0.46** | 0.13 | 0.20 | **0.40** | 0.36 | -0.09 | 0.42 | 0.01 | -0.09 | 0.36 | 0.02 | 0.41 | 0.00 | 0.35 | -0.08 | -0.01 | **0.46** | -0.03 | -0.03 |
| Fear of blood/injection | **0.46** | 0.24 | 0.30 | 0.46 | -0.15 | 0.36 | 0.10 | 0.36 | 0.41 | -0.15 | 0.01 | -0.07 | **0.59** | 0.28 | -0.01 | -0.03 | 0.01 | 0.19 | 0.17 | **0.63** | 0.04 |
| Fear of injury | 0.31 | 0.28 | 0.09 | **0.43** | -0.10 | 0.14 | 0.21 | 0.30 | 0.19 | -0.20 | 0.00 | 0.07 | **0.51** | 0.06 | -0.05 | 0.11 | -0.02 | 0.21 | -0.02 | **0.49** | 0.00 |
| Fear of doctor | 0.32 | 0.29 | 0.09 | **0.44** | -0.10 | 0.15 | -0.03 | **0.44** | 0.18 | -0.03 | 0.25 | -0.12 | **0.39** | 0.12 | 0.01 | -0.08 | 0.26 | 0.13 | -0.01 | **0.43** | 0.06 |
| Fear of animal | 0.33 | 0.14 | 0.23 | 0.30 | -0.11 | 0.27 | 0.03 | 0.26 | 0.30 | -0.08 | 0.02 | -0.09 | **0.40** | 0.21 | 0.02 | -0.07 | 0.00 | 0.19 | 0.17 | **0.35** | 0.04 |
| Arguments with adults | **0.67** | 0.43 | 0.36 | -0.14 | **0.74** | 0.18 | 0.08 | 0.00 | 0.08 | **0.76** | 0.04 | -0.02 | -0.02 | 0.04 | **0.81** | -0.05 | 0.02 | 0.02 | 0.05 | -0.05 | **0.82** |
| Loosing temper | **0.54** | 0.31 | 0.33 | -0.11 | **0.57** | 0.18 | 0.10 | -0.03 | 0.11 | **0.55** | -0.11 | -0.04 | 0.10 | 0.03 | **0.67** | -0.04 | -0.10 | 0.01 | 0.01 | 0.10 | **0.69** |
| Defiance | **0.58** | 0.25 | **0.43** | -0.16 | **0.56** | 0.29 | -0.01 | -0.03 | 0.21 | **0.61** | -0.01 | -0.09 | -0.01 | 0.17 | **0.66** | -0.11 | -0.01 | -0.03 | 0.18 | -0.02 | **0.66** |
| Temper tantrums | **0.61** | **0.46** | 0.27 | -0.03 | **0.66** | 0.12 | 0.16 | 0.05 | 0.04 | **0.61** | 0.09 | 0.09 | -0.01 | 0.02 | **0.65** | 0.07 | 0.09 | 0.00 | 0.01 | -0.02 | **0.65** |
| Angry or resentful | **0.74** | 0.43 | 0.44 | 0.09 | **0.54** | 0.32 | 0.25 | 0.06 | 0.28 | **0.45** | -0.05 | 0.13 | 0.19 | 0.20 | **0.55** | 0.11 | -0.09 | 0.12 | 0.22 | 0.11 | **0.56** |
| Disobedience | **0.72** | 0.29 | **0.54** | -0.04 | 0.50 | 0.43 | 0.04 | 0.04 | 0.36 | **0.53** | 0.02 | -0.04 | 0.07 | 0.32 | **0.57** | -0.04 | 0.04 | -0.01 | 0.32 | 0.05 | **0.56** |
| Irritability | **0.59** | **0.62** | 0.08 | -0.02 | **0.82** | -0.11 | 0.46 | -0.08 | -0.17 | **0.58** | 0.08 | 0.44 | -0.21 | -0.13 | **0.57** | 0.40 | 0.03 | 0.03 | -0.04 | -0.30 | **0.55** |
| Spiteful vindictive | **0.46** | 0.35 | 0.20 | -0.05 | **0.53** | 0.08 | 0.16 | -0.01 | 0.02 | **0.47** | -0.01 | 0.09 | 0.01 | -0.02 | **0.52** | 0.09 | 0.02 | -0.06 | -0.05 | 0.03 | **0.54** |
| Rule breaking | **0.73** | 0.23 | **0.61** | 0.00 | 0.40 | 0.50 | 0.04 | 0.04 | 0.45 | 0.44 | -0.03 | -0.05 | 0.14 | 0.40 | 0.49 | -0.04 | 0.02 | -0.07 | 0.35 | 0.18 | **0.50** |
| Touchy or easily annoyed | **0.62** | **0.44** | 0.30 | 0.10 | **0.52** | 0.18 | 0.26 | 0.06 | 0.14 | **0.41** | 0.02 | 0.17 | 0.11 | 0.09 | **0.48** | 0.14 | -0.07 | 0.22 | 0.19 | -0.06 | **0.46** |
| Easily frustrated | **0.66** | 0.43 | 0.35 | 0.09 | **0.52** | 0.23 | 0.32 | 0.01 | 0.20 | 0.38 | -0.06 | 0.23 | 0.13 | 0.14 | **0.46** | 0.19 | -0.13 | 0.17 | 0.21 | 0.01 | **0.46** |
| Teasing | **0.53** | 0.26 | **0.36** | 0.02 | **0.38** | 0.27 | 0.17 | -0.01 | 0.24 | 0.32 | -0.09 | 0.08 | 0.13 | 0.17 | **0.40** | 0.11 | -0.03 | -0.11 | 0.09 | 0.22 | **0.43** |
| Difficulty with transitions | **0.62** | 0.34 | 0.39 | 0.09 | **0.41** | 0.29 | 0.11 | 0.11 | 0.26 | **0.40** | 0.11 | 0.06 | 0.06 | 0.24 | **0.41** | 0.05 | 0.10 | 0.05 | 0.25 | 0.01 | **0.40** |
| Blaming | **0.63** | 0.29 | **0.44** | 0.12 | 0.32 | 0.36 | 0.17 | 0.08 | 0.34 | 0.27 | -0.05 | 0.07 | 0.22 | 0.27 | 0.36 | 0.10 | 0.00 | -0.05 | 0.20 | 0.28 | **0.39** |
| Annoying behavior | **0.70** | 0.36 | **0.47** | 0.12 | 0.42 | 0.37 | 0.23 | 0.06 | **0.35** | 0.34 | 0.02 | 0.17 | 0.11 | 0.31 | 0.37 | 0.19 | 0.07 | -0.08 | 0.25 | 0.16 | **0.38** |
| Abdominal pains present | **0.36** | 0.31 | 0.12 | 0.23 | 0.20 | 0.08 | 0.32 | 0.08 | 0.10 | 0.02 | 0.00 | 0.29 | 0.14 | 0.07 | 0.05 | 0.31 | 0.04 | -0.04 | 0.00 | 0.21 | 0.09 |
| Sleeps with a family member | 0.29 | 0.31 | 0.05 | 0.29 | 0.10 | 0.04 | 0.13 | 0.25 | 0.05 | 0.06 | 0.15 | 0.09 | 0.20 | 0.03 | 0.07 | 0.11 | 0.17 | 0.04 | -0.06 | 0.24 | 0.10 |
| Ever: Scared or anxious about going to daycare or school | 0.02 | **0.50** | -0.46 | **0.37** | 0.18 | -0.46 | 0.15 | **0.39** | -0.45 | 0.12 | **0.44** | 0.16 | 0.03 | -0.38 | 0.02 | 0.16 | 0.34 | 0.28 | -0.33 | -0.14 | 0.01 |
| Ever: Unable to go to daycare/school because worried or upset | 0.11 | **0.62** | -0.49 | **0.50** | 0.19 | -0.48 | 0.33 | 0.43 | -0.45 | 0.02 | 0.41 | 0.32 | 0.15 | -0.40 | -0.05 | 0.32 | 0.29 | 0.33 | -0.35 | -0.03 | -0.05 |
| Rises to check on family members | **0.37** | 0.23 | 0.20 | 0.32 | -0.01 | 0.21 | 0.25 | 0.16 | 0.26 | -0.13 | 0.09 | 0.27 | 0.14 | 0.27 | -0.18 | 0.30 | 0.15 | -0.08 | 0.16 | 0.25 | -0.15 |
| Other fears | **0.36** | **0.39** | 0.06 | 0.31 | 0.19 | 0.03 | **0.36** | 0.15 | 0.06 | -0.01 | -0.04 | 0.26 | 0.31 | -0.03 | 0.10 | 0.27 | -0.08 | 0.17 | -0.05 | 0.25 | 0.14 |
| Fear of storms | **0.41** | 0.33 | 0.17 | 0.33 | 0.10 | 0.16 | 0.23 | 0.23 | 0.18 | 0.00 | 0.06 | 0.15 | 0.30 | 0.12 | 0.07 | 0.17 | 0.06 | 0.10 | 0.05 | 0.31 | 0.10 |
| Fear of dark | **0.37** | 0.30 | 0.15 | 0.29 | 0.10 | 0.13 | 0.20 | 0.20 | 0.15 | 0.02 | 0.03 | 0.11 | 0.30 | 0.08 | 0.10 | 0.13 | 0.01 | 0.14 | 0.05 | 0.27 | 0.13 |
| Fear of clowns | 0.21 | 0.21 | 0.04 | **0.45** | -0.24 | 0.14 | -0.03 | **0.43** | 0.19 | -0.19 | 0.16 | -0.16 | **0.49** | 0.09 | -0.08 | -0.14 | 0.07 | 0.38 | 0.11 | 0.32 | -0.07 |
| Thinking about death | **0.43** | **0.38** | 0.14 | 0.23 | 0.28 | 0.09 | 0.34 | 0.10 | 0.10 | 0.10 | -0.04 | 0.26 | 0.23 | 0.03 | 0.19 | 0.28 | 0.00 | -0.01 | -0.06 | 0.29 | 0.23 |
| Death themes in play | **0.48** | **0.44** | 0.13 | 0.02 | **0.57** | 0.00 | 0.43 | -0.11 | -0.02 | 0.34 | -0.03 | 0.42 | -0.10 | -0.02 | 0.34 | 0.42 | 0.01 | -0.14 | -0.05 | -0.04 | 0.36 |
| Reduced appetite present | 0.30 | 0.28 | 0.08 | 0.25 | 0.10 | 0.08 | 0.13 | 0.20 | 0.09 | 0.06 | 0.17 | 0.11 | 0.13 | 0.09 | 0.04 | 0.09 | 0.09 | 0.21 | 0.15 | -0.01 | 0.03 |
| Weight loss present | 0.34 | 0.34 | 0.07 | 0.32 | 0.11 | 0.06 | 0.04 | 0.32 | 0.07 | 0.14 | 0.32 | 0.05 | 0.09 | 0.10 | 0.06 | 0.06 | 0.30 | 0.09 | 0.06 | 0.07 | 0.07 |
| Excessive appetite present | 0.28 | 0.05 | 0.27 | 0.19 | -0.09 | 0.29 | -0.03 | 0.17 | 0.31 | -0.04 | 0.10 | -0.03 | 0.14 | 0.30 | -0.06 | -0.01 | 0.14 | -0.03 | 0.23 | 0.20 | -0.05 |
| Increased need for sleep | **0.38** | 0.29 | 0.16 | 0.29 | 0.09 | 0.15 | **0.39** | 0.08 | 0.20 | -0.14 | -0.08 | 0.34 | 0.26 | 0.13 | -0.06 | 0.38 | -0.03 | -0.06 | 0.02 | 0.36 | -0.01 |
| Difficulty restoring emotional equilibrium | **0.49** | **0.43** | 0.16 | 0.09 | **0.48** | 0.05 | 0.24 | 0.07 | 0.02 | **0.38** | 0.16 | 0.22 | -0.07 | 0.04 | **0.35** | 0.18 | 0.06 | 0.20 | 0.18 | -0.27 | 0.31 |

Notes: Bold indicates primary loadings (≥0.35) with at least 0.10 difference from the second largest loading. Factor correlations ≥ 0.11 were statistically significant (*p*<0.05). The 1-factor solution was used to model the p factor, whereas the 6-factor solution (the most differentiated interpretable solution) was used to model the specific factors.

Abbreviations: Distr=Distress; Ext=Externalizing; F=Factor; Inatt-Hyp=Inattention-hyperactivity; Int=Internalizing; p=General psychopathology; Opp =Oppositionality; Sep Anx=Separation anxiety; Soc Anx= Social anxiety.

**Table S2. Correlations between factors from different exploratory factor solutions**

|  |  | **1-factor** | **2-factor** | | **3-factor** | | | **4-factor** | | | | **5-factor** | | | | | **6-factor** | | | | | |
| --- | --- | --- | --- | --- | --- | --- | --- | --- | --- | --- | --- | --- | --- | --- | --- | --- | --- | --- | --- | --- | --- | --- |
|  |  | p | Int | Ext | Fear | Distr-Opp | Inatt-Hyp | Distr | Fear | Inatt-Hyp | Opp | Sep Anx | Distr | Fear | Inatt-Hyp | Opp | Distr | Sep Anx | Soc Anx | Inatt-Hyp | Fear | Opp |
| **1-factor** | p | 1 |  |  |  |  |  |  |  |  |  |  |  |  |  |  |  |  |  |  |  |  |
| **2-factor** | Int | .841^**^ | 1 |  |  |  |  |  |  |  |  |  |  |  |  |  |  |  |  |  |  |  |
|  | Ext | .920^**^ | .572^**^ | 1 |  |  |  |  |  |  |  |  |  |  |  |  |  |  |  |  |  |  |
| **3-factor** | Fear | .675^**^ | .827^**^ | .428^**^ | 1 |  |  |  |  |  |  |  |  |  |  |  |  |  |  |  |  |  |
|  | Distr-Opp | .844^**^ | .896^**^ | .660^**^ | .510^**^ | 1 |  |  |  |  |  |  |  |  |  |  |  |  |  |  |  |  |
|  | Inatt-Hyp | .859^**^ | .463^**^ | .980^**^ | .416^**^ | .512^**^ | 1 |  |  |  |  |  |  |  |  |  |  |  |  |  |  |  |
| **4-factor** | Distr | .762^**^ | .878^**^ | .536^**^ | .751^**^ | .791^**^ | .436^**^ | 1 |  |  |  |  |  |  |  |  |  |  |  |  |  |  |
|  | Fear | .533^**^ | .716^**^ | .291^**^ | .918^**^ | .382^**^ | .297^**^ | .482^**^ | 1 |  |  |  |  |  |  |  |  |  |  |  |  |  |
|  | Inatt-Hyp | .856^**^ | .478^**^ | .962^**^ | .484^**^ | .483^**^ | .991^**^ | .491^**^ | .337^**^ | 1 |  |  |  |  |  |  |  |  |  |  |  |  |
|  | Opp | .778^**^ | .742^**^ | .674^**^ | .308^**^ | .925^**^ | .539^**^ | .512^**^ | .296^**^ | .467^**^ | 1 |  |  |  |  |  |  |  |  |  |  |  |
| **5-factor** | Sep Anx | .446^**^ | .657^**^ | .200^**^ | .719^**^ | .429^**^ | .167^**^ | .344^**^ | .846^**^ | .169^**^ | .427^**^ | 1 |  |  |  |  |  |  |  |  |  |  |
|  | Distr | .737^**^ | .851^**^ | .513^**^ | .719^**^ | .775^**^ | .412^**^ | .974^**^ | .443^**^ | .465^**^ | .503^**^ | .404^**^ | 1 |  |  |  |  |  |  |  |  |  |
|  | Fear | .572^**^ | .637^**^ | .414^**^ | .841^**^ | .351^**^ | .433^**^ | .618^**^ | .778^**^ | .500^**^ | .170^**^ | .351^**^ | .481^**^ | 1 |  |  |  |  |  |  |  |  |
|  | Inatt-Hyp | .854^**^ | .466^**^ | .968^**^ | .444^**^ | .495^**^ | .992^**^ | .462^**^ | .305^**^ | .992^**^ | .500^**^ | .194^**^ | .457^**^ | .416^**^ | 1 |  |  |  |  |  |  |  |
|  | Opp | .833^**^ | .791^**^ | .723^**^ | .380^**^ | .951^**^ | .591^**^ | .611^**^ | .327^**^ | .533^**^ | .979^**^ | .361^**^ | .569^**^ | .316^**^ | .546^**^ | 1 |  |  |  |  |  |  |
| **6-factor** | Distr | .728^**^ | .851^**^ | .499^**^ | .731^**^ | .764^**^ | .400^**^ | .973^**^ | .458^**^ | .455^**^ | .488^**^ | .411^**^ | .998^**^ | .495^**^ | .445^**^ | .555^**^ | 1 |  |  |  |  |  |
|  | Sep Anx | .517^**^ | .644^**^ | .321^**^ | .685^**^ | .455^**^ | .295^**^ | .323^**^ | .807^**^ | .288^**^ | .486^**^ | .970^**^ | .393^**^ | .320^**^ | .323^**^ | .411^**^ | .405^**^ | 1 |  |  |  |  |
|  | Soc Anx | .308^**^ | .556^**^ | .056^*^ | .712^**^ | .272^**^ | 0.044 | .459^**^ | .729^**^ | .094^**^ | .124^**^ | .544^**^ | .379^**^ | .681^**^ | 0.036 | .202^**^ | .367^**^ | .367^**^ | 1 |  |  |  |
|  | Inatt-Hyp | .884^**^ | .521^**^ | .972^**^ | .487^**^ | .542^**^ | .987^**^ | .516^**^ | .341^**^ | .990^**^ | .532^**^ | .217^**^ | .499^**^ | .464^**^ | .991^**^ | .588^**^ | .485^**^ | .320^**^ | .140^**^ | 1 |  |  |
|  | Fear | .508^**^ | .415^**^ | .484^**^ | .627^**^ | .206^**^ | .534^**^ | .425^**^ | .565^**^ | .589^**^ | .093^**^ | .149^**^ | .313^**^ | .831^**^ | .525^**^ | .213^**^ | .344^**^ | .246^**^ | .179^**^ | .501^**^ | 1 |  |
|  | Opp | .843^**^ | .805^**^ | .728^**^ | .403^**^ | .955^**^ | .597^**^ | .628^**^ | .346^**^ | .542^**^ | .976^**^ | .371^**^ | .585^**^ | .337^**^ | .554^**^ | .999^**^ | .573^**^ | .424^**^ | .202^**^ | .593^**^ | .245^**^ | 1 |

Notes: *******p*<.01; ******p*<.05. Correlations between factors within the same factor solution (e.g., between internalizing and externalizing factors from the 2-factor solution) are highlighted in grey. Other correlations are between factors across different factor solutions.

Abbreviations: Distr=Distress; Ext=Externalizing; F=Factor; Inatt-Hyp=Inattention-hyperactivity; Int=Internalizing; p=General psychopathology; Opp=Oppositionality; Sep Anx=Separation anxiety; Soc Anx= Social anxiety.

**Table S3. Correlations between specific factors from the exploratory 6-factor model (with and without regressing the effect of the general p factor out) and confirmatory bifactor model.**

|  | **Bifactor model** | | | | | |
| --- | --- | --- | --- | --- | --- | --- |
|  | Distress | Separation anxiety | Social anxiety | Inattention-hyperactivity | Fear | Oppositionality |
| **Exploratory 6-factor model** | | | | | | |
| Distress | .706^**^ | .090^**^ | .085^**^ | -.079^**^ | .013 | .052 |
| Separation anxiety | .097^**^ | .809^**^ | .206^**^ | -0.017 | .117^**^ | .051 |
| Social anxiety | .155^**^ | .299^**^ | .762^**^ | -.204^**^ | .256^**^ | -.004 |
| Inattention-hyperactivity | -.074^**^ | -.094^**^ | -.052 | .622^**^ | .085^**^ | .016 |
| Fear | .007 | -0.051 | .151^**^ | .025 | .663^**^ | -.284^**^ |
| Oppositionality | .053 | .009 | -.062^*^ | -.058^*^ | -.087^**^ | .648^**^ |
| **Exploratory 6-factor model (regressing p factor out)** | | | | | | |
| Distress | .880^**^ | .071^*^ | .086^**^ | -.397^**^ | -.069^*^ | -.183^**^ |
| Separation anxiety | 0.028 | .911^**^ | .219^**^ | -.180^**^ | .086^**^ | -.088^**^ |
| Social anxiety | .118^**^ | .296^**^ | .790^**^ | -.300^**^ | .242^**^ | -.084^**^ |
| Inattention-hyperactivity | -.425^**^ | -.309^**^ | -.178^**^ | .830^**^ | .024 | -.429^**^ |
| Fear | -.075^**^ | -.093^**^ | .154^**^ | -.127^**^ | .720^**^ | -.474^**^ |
| Oppositionality | -.123^**^ | -.071^*^ | -.171^**^ | -.524^**^ | -.293^**^ | .823^**^ |

Notes: *******p*<.01; ******p*<.05. Correlations between equivalent factors (i.e., loading on the same items) from different models (i.e., between the Distress factors from bifactor model and exploratory 6-factor model) are highlighted in grey.

**Table S4. Full results of hierarchical regression models to predict preadolescent psychiatric disorders and functional impairment.**

|  |  |  | **Preschool age, sex**  **(block 1)** | **Preschool DSM diagnoses**  **(block 2)** | **Preschool general (p) factor**  **(block 3)** | **Preschool specific factors**  **(block 4)** |
| --- | --- | --- | --- | --- | --- | --- |
| **GAD** | MAPS+PDS | R^2^ | .01 | .03 | .05 | .20 |
|  |  | R^2^ change |  | .03 | .02 | .15 |
|  |  | p |  | .05 | .11 | **<.01** |
|  |  | AUC | .57 | .66 | .68 | .80 |
|  |  | AUC change |  | .09 | .02 | .12 |
|  | SBTS | R^2^ | .02 | .02 | .06 | .07 |
|  |  | R^2^ change |  | .00 | .04 | .01 |
|  |  | p | .34 | .80 | **.02** | .87 |
|  |  | AUC | .60 | .60 | .68 | .68 |
|  |  | AUC change |  | <.01 | .08 | <.01 |
|  | SBTS (excluding preschool cases) | R^2^ | .02 |  | .04 | .11 |
|  |  | R^2^ change |  |  | .02 | .07 |
|  |  | p |  |  | .15 | .34 |
|  |  | AUC | .60 |  | .65 | .65 |
|  |  | AUC change |  |  | .05 | <.01 |
| **Separation anxiety disorder** | MAPS+PDS | R^2^ | .01 | .05 | .17 | .24 |
|  |  | R^2^ change |  | .04 | .12 | .07 |
|  |  | p |  | **.03** | **<.01** | .13 |
|  |  | AUC | .59 | .67 | .78 | .85 |
|  |  | AUC change |  | .08 | .11 | .07 |
|  | SBTS | R^2^ | .03 | .07 | .08 | .10 |
|  |  | R^2^ change |  | .04 | .02 | .02 |
|  |  | p |  | **.04** | .18 | .88 |
|  |  | AUC | .64 | .70 | .73 | .78 |
|  |  | AUC change |  | .06 | .03 | .05 |
|  | SBTS (excluding preschool cases) | R^2^ | .04 |  | .05 | .10 |
|  |  | R^2^ change |  |  | .01 | .05 |
|  |  | p |  |  | .40 | .79 |
|  |  | AUC | .68 |  | .70 | .78 |
|  |  | AUC change |  |  | .02 | .08 |
| **ADHD** | MAPS+PDS | R^2^ | .03 | .04 | .19 | .21 |
|  |  | R^2^ change |  | .02 | .15 | .02 |
|  |  | p |  | **.04** | **<.01** | .33 |
|  |  | AUC | .60 | .63 | .75 | .77 |
|  |  | AUC change |  | .03 | .12 | .02 |
|  | SBTS | R^2^ | .06 | .07 | .15 | .20 |
|  |  | R^2^ change |  | .02 | .08 | .05 |
|  |  | p |  | **.05** | **<.01** | .12 |
|  |  | AUC | .65 | .69 | .74 | .76 |
|  |  | AUC change |  | .04 | .05 | .02 |
|  | SBTS (excluding preschool cases) | R^2^ | .07 |  | .12 | .14 |
|  |  | R^2^ change |  |  | .05 | .02 |
|  |  | p |  |  | **<.01** | .87 |
|  |  | AUC | .69 |  | .73 | .73 |
|  |  | AUC change |  |  | .04 | <.01 |
| **ODD** | MAPS+PDS | R^2^ | .01 | .05 | .15 | .21 |
|  |  | R^2^ change |  | .04 | .10 | .06 |
|  |  | p |  | **<.01** | **<.01** | **.02** |
|  |  | AUC | .57 | .66 | .73 | .77 |
|  |  | AUC change |  | .09 | .07 | .04 |
|  | SBTS | R^2^ | .06 | .08 | .09 | .24 |
|  |  | R^2^ change |  | .02 | .01 | .15 |
|  |  | p |  | .22 | .28 | **.01** |
|  |  | AUC | .68 | .72 | .72 | .87 |
|  |  | AUC change |  | .04 | <.01 | .15 |
|  | SBTS (excluding preschool cases) | R^2^ | .07 |  | .07 | .26 |
|  |  | R^2^ change |  |  | .00 | .19 |
|  |  | p |  |  | .58 | .07 |
|  |  | AUC | .71 |  | .72 | .89 |
|  |  | AUC change |  |  | .01 | .17 |
| **Depressive disorder** | MAPS+PDS | R^2^ | .03 | .04 | .10 | .23 |
|  |  | R^2^ change |  | .01 | .06 | .13 |
|  |  | p |  | .13 | **<.01** | **<.01** |
|  |  | AUC | .63 | .64 | .71 | .79 |
|  |  | AUC change |  | .01 | .07 | .08 |
| **CD** | MAPS+PDS | R^2^ | .13 | .22 | .37 | .52 |
|  |  | R^2^ change |  | .09 | .15 | .15 |
|  |  | p |  | **<.01** | **<.01** | **.01** |
|  |  | AUC | .81 | .87 | .91 | .96 |
|  |  | AUC change |  | .06 | .04 | .05 |
|  |  |  | **Preschool age, sex**  **(block 1)** | **Preschool functioning**  **(block 2)** | **Preschool general (p) factor**  **(block 3)** | **Preschool specific factors**  **(block 4)** |
| **Functional impairment** | MAPS+PDS | R^2^ | .02 | .10 | .17 | .20 |
|  |  | R^2^ change |  | .08 | .07 | .03 |
|  |  | p |  | **<.01** | **<.01** | **<.01** |
|  | SBTS | R^2^ | .01 | .08 | .09 | .14 |
|  |  | R^2^ change |  | .07 | .01 | .05 |
|  |  | p |  | **<.01** | **.02** | **<.01** |
|  |  |  | **Preschool age, sex**  **(block 1)** | **Preschool functioning**  **(block 2)** | **Preschool DSM diagnoses**  **(block 3)** |  |
| **Functional impairment** | MAPS+PDS | R^2^ | .02 | .10 | .11 |  |
|  |  | R^2^ change |  | .08 | .01 |  |
|  |  | p |  | **<.01** | .13 |  |
|  | SBTS | R^2^ | .01 | .08 | .09 |  |
|  |  | R^2^ change |  | .07 | .01 |  |
|  |  | p |  | **<.01** | **.02** |  |

Notes: Bold indicates significant *p* values (*p*<.05). Current preadolescent diagnoses were examined in MAPS+PDS, whereas diagnoses in the interval between age 9 and 12 assessments were examined in SBTS. Depressive disorders and CD were investigated only in MAPS+PDS as only one and no participants in SBTS respectively met criteria for these conditions. Results for preadolescence disorders in SBTS are reported on the total sample, as well as after removing participants with preschool psychiatric diagnoses. In analyses of preadolescent diagnoses (binary outcomes), R^2^ represent Nagelkerke pseudo-R^2^. AUCs are reported to provide an additional metric of variance explained, but should be interpreted cautiously, especially for disorders showing low rates in these samples (see Table 1 in the main text).

Abbreviations: ADHD, attention-deficit/hyperactivity disorder; AUC, area under the curve; CD, conduct disorder; GAD, generalized anxiety disorder; MAPS, Multidimensional Assessment of Preschoolers Study; ODD, oppositional defiant disorder; PDS, Preschool Depression Study; SBTS, Stony Brook Temperament Study.

**Table S5. Full results of hierarchical regression models to predict preadolescent psychiatric disorders and functional impairment separately in MAPS and PDS.**

|  |  |  | **Preschool age, sex**  **(block 1)** | **Preschool DSM diagnoses**  **(block 2)** | **Preschool general (p) factor**  **(block 3)** | **Preschool specific factors**  **(block 4)** |
| --- | --- | --- | --- | --- | --- | --- |
| **GAD** | MAPS | R^2^ | <.01 | <.01 | <.01 | .28 |
|  |  | R^2^ change |  | <.01 | <.01 | .27 |
|  |  | p |  | .93 | .74 | **.04** |
|  |  | AUC | .55 | .54 | .54 | .87 |
|  |  | AUC change |  | -.01 | <.01 | .33 |
|  | PDS | R^2^ | .03 | .14 | .14 | .24 |
|  |  | R^2^ change |  | .11 | <.01 | .10 |
|  |  | p |  | **<.01** | .71 | .12 |
|  |  | AUC | .61 | .74 | .74 | .81 |
|  |  | AUC change |  | .13 | <.01 | .07 |
| **Separation anxiety disorder** | MAPS | R^2^ | .06 | .07 | .30 | .41 |
|  |  | R^2^ change |  | .01 | .23 | .11 |
|  |  | p |  | .26 | **<.01** | .15 |
|  |  | AUC | .68 | .71 | .87 | .93 |
|  |  | AUC change |  | .03 | .16 | .06 |
|  | PDS | R^2^ | .02 | .08 | .08 | .16 |
|  |  | R^2^ change |  | .06 | <.01 | .08 |
|  |  | p |  | .09 | .55 | .69 |
|  |  | AUC | .61 | .72 | .73 | .80 |
|  |  | AUC change |  | .11 | .02 | .07 |
| **ADHD** | MAPS | R^2^ | <.01 | <.01 | .10 | .13 |
|  |  | R^2^ change |  | <.01 | .09 | .03 |
|  |  | p |  | .85 | **<.01** | .66 |
|  |  | AUC | .57 | .55 | .70 | .73 |
|  |  | AUC change |  | -.02 | .15 | .03 |
|  | PDS | R^2^ | .06 | .14 | .30 | .36 |
|  |  | R^2^ change |  | .08 | .16 | .06 |
|  |  | p |  | **<.01** | **<.01** | .16 |
|  |  | AUC | .65 | .73 | .79 | .83 |
|  |  | AUC change |  | .08 | .06 | .04 |
| **ODD** | MAPS | R^2^ | <.01 | .02 | .15 | .22 |
|  |  | R^2^ change |  | .02 | .13 | .07 |
|  |  | p |  | .14 | **<.01** | .15 |
|  |  | AUC | .54 | .57 | .72 | .78 |
|  |  | AUC change |  | .03 | .15 | .06 |
|  | PDS | R^2^ | .07 | .16 | .20 | .30 |
|  |  | R^2^ change |  | .09 | .04 | .10 |
|  |  | p |  | **<.01** | .05 | .08 |
|  |  | AUC | .67 | .76 | .77 | .81 |
|  |  | AUC change |  | .10 | .01 | .04 |
| **Depressive disorder** | MAPS | R^2^ | .15 | .20 | .21 | 1.00 |
|  |  | R^2^ change |  | .05 | .01 | .79 |
|  |  | p |  | .31 | .59 | **<.01** |
|  |  | AUC | .87 | .91 | .91 | 1.00 |
|  |  | AUC change |  | .04 | <.01 | .09 |
|  | PDS | R^2^ | .07 | .15 | .18 | .23 |
|  |  | R^2^ change |  | .08 | .03 | .05 |
|  |  | p |  | **<.01** | **.03** | .20 |
|  |  | AUC | .66 | .73 | .74 | .77 |
|  |  | AUC change |  | .06 | .01 | .03 |
| **CD** | MAPS | R^2^ | .09 | .14 | .39 | .45 |
|  |  | R^2^ change |  | .05 | .25 | .06 |
|  |  | p |  | .13 | **<.01** | .84 |
|  |  | AUC | .81 | .86 | .93 | .94 |
|  | PDS | R^2^ | .23 | .39 | .44 | .71 |
|  |  | R^2^ change |  | .16 | .05 | .27 |
|  |  | p |  | **<.01** | .08 | **<.01** |
|  |  | AUC | .84 | .92 | .92 | .99 |
|  |  | AUC change |  | .08 | <.01 | .07 |
|  |  |  | **Preschool age, sex**  **(block 1)** | **Preschool functioning**  **(block 2)** | **Preschool general (p) factor**  **(block 3)** | **Preschool specific factors**  **(block 4)** |
| **Functional impairment** | MAPS | R^2^ | <.01 | .08 | .15 | .17 |
|  |  | R^2^ change |  | .08 | .07 | .02 |
|  |  | p |  | **<.01** | **<.01** | .30 |
|  | PDS | R^2^ | .06 | .19 | .24 | .29 |
|  |  | R^2^ change |  | .13 | .05 | .05 |
|  |  | p |  | **<.01** | **<.01** | **.03** |
|  |  |  | **Preschool age, sex**  **(block 1)** | **Preschool functioning**  **(block 2)** | **Preschool DSM diagnoses**  **(block 3)** |  |
| **Functional impairment** | MAPS | R^2^ | <.01 | .08 | .10 |  |
|  |  | R^2^ change |  | .08 | .01 |  |
|  |  | p |  | **<.01** | .05 |  |
|  | PDS | R^2^ | .06 | .19 | .20 |  |
|  |  | R^2^ change |  | .13 | .01 |  |
|  |  | p |  | **<.01** | .08 |  |

Notes: Bold indicates significant *p* values (*p*<.05). Current preadolescent diagnoses were examined. In analyses of preadolescent diagnoses (binary outcomes), R^2^ represent Nagelkerke pseudo-R^2^. AUCs are reported to provide an additional metric of variance explained, but should be interpreted cautiously, especially for disorders showing low rates in these samples (see Table 1 in the main text).

Abbreviations: ADHD, attention-deficit/hyperactivity disorder; AUC, area under the curve; CD, conduct disorder; GAD, generalized anxiety disorder; MAPS, Multidimensional Assessment of Preschoolers Study; ODD, oppositional defiant disorder; PDS, Preschool Depression Study.

**Table S6. Results of hierarchical regression models to predict preadolescent psychiatric disorders and functional impairment from 2-factor, 3-factor, 4-factor and 5-factor solutions over and over a model including a p factor and covariates.**

|  |  |  | **Model 1: Preschool age, sex, preschool DSM diagnoses, preschool general (p) factor** | **Model 1 vs. Model 2 (Model 1 + 2-factor solution)** | **Model 1 vs. Model 3 (Model 1 + 3-factor solution)** | **Model 1 vs. Model 4 (Model 1 + 4-factor solution)** | **Model 1 vs. Model 5 (Model 1 + 5-factor solution)** | **Model 1 vs. Model 6 (Model 1 + 6-factor solution)** |
| --- | --- | --- | --- | --- | --- | --- | --- | --- |
| **GAD** | MAPS+PDS | R^2^ | .05 | .14 | .15 | .16 | .19 | .20 |
|  |  | R^2^ change |  | .09 | .10 | .11 | .14 | .15 |
|  |  | p |  | **<.01** | **<.01** | **<.01** | **<.01** | **<.01** |
|  |  | AUC | .68 | .78 | .78 | .78 | .80 | .80 |
|  |  | AUC change |  | .10 | .10 | .10 | .12 | .12 |
|  | SBTS | R^2^ | .06 | .06 | .06 | .08 | .08 | .07 |
|  |  | R^2^ change |  | <.01 | <.01 | .02 | .02 | .01 |
|  |  | p |  | .99 | .99 | .51 | .76 | .87 |
|  |  | AUC | .68 | .68 | .68 | .69 | .68 | .68 |
|  |  | AUC change |  | <.01 | <.01 | .01 | <.01 | <.01 |
|  | SBTS (excluding preschool cases) | R^2^ | .04 | .05 | .05 | .10 | .10 | .11 |
|  |  | R^2^ change |  | .01 | .01 | .06 | .06 | .07 |
|  |  | p |  | .60 | .76 | .23 | .33 | .34 |
|  |  | AUC | .65 | .66 | .65 | .67 | .67 | .65 |
|  |  | AUC change |  | .01 | <.01 | .02 | .02 | <.01 |
| **Separation anxiety disorder** | MAPS+PDS | R^2^ | .17 | .21 | .23 | .23 | .24 | .24 |
|  |  | R^2^ change |  | .04 | .06 | .06 | .07 | .07 |
|  |  | p |  | .06 | .05 | .10 | .11 | .13 |
|  |  | AUC | .78 | .83 | .83 | .84 | .84 | .85 |
|  |  | AUC change |  | .05 | .05 | .06 | .06 | .07 |
|  | SBTS | R^2^ | .08 | .09 | .09 | .09 | .10 | .10 |
|  |  | R^2^ change |  |  |  |  |  | .02 |
|  |  | p |  | .71 | .82 | .89 | .85 | .88 |
|  |  | AUC | .73 | .75 | .76 | .75 | .77 | .78 |
|  |  | AUC change |  | .02 | .03 | .02 | .04 | .05 |
|  | SBTS (excluding preschool cases) | R^2^ | .05 | .08 | .09 | .09 | .11 | .10 |
|  |  | R^2^ change |  | .03 | .04 | .04 | .06 | .05 |
|  |  | p |  | .44 | .46 | .67 | .62 | .79 |
|  |  | AUC | .70 | .74 | .76 | .76 | .80 | .78 |
|  |  | AUC change |  | .04 | .06 | .06 | .10 | .08 |
| **ADHD** | MAPS+PDS | R^2^ | .19 | .19 | .20 | .20 | .20 | .21 |
|  |  | R^2^ change |  | <.01 | .01 | .01 | .01 | .02 |
|  |  | p |  | .72 | .23 | .34 | .46 | .33 |
|  |  | AUC | .75 | .75 | .76 | .76 | .76 | .77 |
|  |  | AUC change |  | <.01 | .01 | .01 | .01 | .02 |
|  | SBTS | R^2^ | .15 | .17 | .19 | .19 | .20 | .20 |
|  |  | R^2^ change |  | .02 | .04 | .04 | .05 | .05 |
|  |  | p |  | .11 | **.03** | .06 | .07 | .12 |
|  |  | AUC | .74 | .75 | .76 | .76 | .76 | .76 |
|  |  | AUC change |  | .01 | .02 | .02 | .02 | .02 |
|  | SBTS (excluding preschool cases) | R^2^ | .12 | .13 | .14 | .14 | .14 | .14 |
|  |  | R^2^ change |  | .01 | .02 | .02 | .02 | .02 |
|  |  | p |  | .66 | .52 | .65 | .80 | .87 |
|  |  | AUC | .73 | .74 | .73 | .73 | .73 | .73 |
|  |  | AUC change |  | .01 | <.01 | <.01 | <.01 | <.01 |
| **ODD** | MAPS+PDS | R^2^ | .15 | .16 | .17 | .20 | .20 | .21 |
|  |  | R^2^ change |  | .01 | .02 | .05 | .05 | .06 |
|  |  | p |  | .53 | .12 | **.02** | **.04** | **.02** |
|  |  | AUC | .73 | .74 | .75 | .76 | .76 | .77 |
|  |  | AUC change |  | .01 | .02 | .03 | .03 | .04 |
|  | SBTS | R^2^ | .09 | .12 | .17 | .20 | .23 | .24 |
|  |  | R^2^ change |  | .03 | .08 | .11 | .14 | .16 |
|  |  | p |  | .22 | **.04** | **.02** | **.01** | **.01** |
|  |  | AUC | .72 | .76 | .83 | .86 | .86 | .87 |
|  |  | AUC change |  | .04 | .11 | .14 | .14 | .15 |
|  | SBTS (excluding preschool cases) | R^2^ | .07 | .10 | .16 | .21 | .23 | .26 |
|  |  | R^2^ change |  | .03 | .09 | .14 | .16 | .19 |
|  |  | p |  | .50 | .14 | .09 | .08 | .07 |
|  |  | AUC | .72 | .74 | .83 | .86 | .87 | .89 |
|  |  | AUC change |  | .02 | .11 | .14 | .15 | .17 |
| **Depressive disorder** | MAPS+PDS | R^2^ | .10 | .13 | .17 | .19 | .23 | .23 |
|  |  | R^2^ change |  | .03 | .07 | .09 | .13 | .13 |
|  |  | p |  | .06 | **<.01** | **<.01** | **<.01** | **<.01** |
|  |  | AUC | .71 | .74 | .74 | .76 | .78 | .79 |
|  |  | AUC change |  | .03 | .03 | .05 | .07 | .08 |
| **CD** | MAPS+PDS | R^2^ | .37 | .40 | .45 | .46 | .47 | .52 |
|  |  | R^2^ change |  | .03 | .08 | .08 | .10 | .15 |
|  |  | p |  | .23 | **.04** | .06 | .05 | **.01** |
|  |  | AUC | .91 | .93 | .95 | .95 | .96 | .96 |
|  |  | AUC change |  | .02 | .04 | .04 | .05 | .05 |
|  |  |  | **Model 1: Preschool age, sex, preschool functioning, preschool general (p) factor** | **Model 1 vs. Model 2 (Model 1 + 2-factor solution)** | **Model 1 vs. Model 3 (Model 1 + 3-factor solution)** | **Model 1 vs. Model 4 (Model 1 + 4-factor solution)** | **Model 1 vs. Model 5 (Model 1 + 5-factor solution)** | **Model 1 vs. Model 6 (Model 1 + 6-factor solution)** |
| **Functional impairment** | MAPS+PDS | R^2^ | .17 | .18 | .20 | .21 | .20 | .20 |
|  |  | R^2^ change |  | .01 | .03 | .04 | .03 | .03 |
|  |  | p |  | .05 | **<.01** | **<.01** | **<.01** | **<.01** |
|  | SBTS | R^2^ | .09 | .09 | .11 | .11 | .11 | .14 |
|  |  | R^2^ change |  | <.01 | .02 | .02 | .02 | .05 |
|  |  | p |  | .65 | **.02** | .05 | **.04** | **<.01** |

Notes: Bold indicates significant *p* values (*p*<.05). Current preadolescent diagnoses were examined in MAPS+PDS, whereas diagnoses in the interval between age 9 and 12 assessments were examined in SBTS. Depressive disorders and CD were investigated only in MAPS+PDS as only one and no participants in SBTS respectively met criteria for these conditions. Results for preadolescence disorders in SBTS are reported on the total sample, as well as after removing participants with preschool psychiatric diagnoses. In analyses of preadolescent diagnoses (binary outcomes), R^2^ represent Nagelkerke pseudo-R^2^. AUCs are reported to provide an additional metric of variance explained, but should be interpreted cautiously, especially for disorders showing low rates in these samples (see Table 1 in the main text). Results for Models 1 and 6 are also reported in Table S3, but repeated here to allow comparison between models with a varying number of factors.

Abbreviations: ADHD, attention-deficit/hyperactivity disorder; AUC, area under the curve; CD, conduct disorder; GAD, generalized anxiety disorder; MAPS, Multidimensional Assessment of Preschoolers Study; ODD, oppositional defiant disorder; PDS, Preschool Depression Study; SBTS, Stony Brook Temperament Study.

**Table S7. Bivariate associations of preschool psychopathology dimensions (rows) with preadolescence psychiatric and functional outcomes (columns) separately in MAPS and PDS.**

|  |  | **GAD** | **Separation anxiety disorder** | **ADHD** | **ODD** | **Depressive**  **disorder** | **CD** | **Functional impairment** |
| --- | --- | --- | --- | --- | --- | --- | --- | --- |
|  |  | OR | OR | OR | OR | OR | OR | β |
| **General (p) factor** | MAPS | 1.20 | 5.90** | 2.24** | 3.25** | .92 | 8.97** | .40** |
|  | PDS | 2.06* | 2.24 | 4.27** | 2.81** | 2.30** | 4.34** | .40** |
| **Distress** | MAPS | 2.61* | 4.33** | 1.51 | 1.60* | 2.16 | 4.49** | .29** |
|  | PDS | 2.61 | 1.72 | 2.06** | 1.76* | 1.49* | 3.64** | .24** |
| **Separation anxiety** | MAPS | 1.07 | 2.13** | 1.16 | 1.47* | .32 | 1.88 | .11 |
|  | PDS | 2.03** | 1.88 | 1.54* | 2.07** | 1.62** | 3.54** | .16* |
| **Social anxiety** | MAPS | 3.00* | 3.68** | 1.11 | 1.07 | 1.44 | 1.14 | .18* |
|  | PDS | 1.16 | 1.56 | 1.16 | 1.08 | 1.02 | 1.03 | <.01 |
| **Fear** | MAPS | .93 | 2.26* | 1.38 | 1.70* | 2.40 | 2.25 | .22** |
|  | PDS | 1.19 | 1.10 | .80 | 1.03 | .87 | 1.53 | -.01 |
| **Inattention-hyperactivity** | MAPS | .57 | 3.08** | 2.25** | 2.15** | 1.45 | 5.22* | .34** |
|  | PDS | 1.50 | 1.72 | 3.09** | 1.80* | 2.02** | 2.05 | .35** |
| **Oppositionality** | MAPS | 1.27 | 3.37** | 1.53 | 3.42** | .10 | 5.12** | .27** |
|  | PDS | 1.30 | 1.86 | 2.99** | 3.18** | 1.86** | 4.28** | .40** |

Notes: **p*<.05, ***p*<.01. All analyses were run on standardized variables and controlled for sex and preschool age.

Abbreviations: ADHD, attention-deficit/hyperactivity disorder; CD, conduct disorder; GAD, generalized anxiety disorder; MAPS, Multidimensional Assessment of Preschoolers Study; ODD, oppositional defiant disorder; OR, odds ratio; PDS, Preschool Depression Study.

**Supplementary references**

Egger, H. L., Erkanli, A., Keeler, G., Potts, E., Walter, B. K., & Angold, A. (2006). Test-Retest Reliability of the Preschool Age Psychiatric Assessment (PAPA). *Journal of the American Academy of Child and Adolescent Psychiatry*, *45*(5), 538–549. doi: 10.1097/01.chi.0000205705.71194.b8

Floyd, F. J., & Widaman, K. F. (1995). Factor analysis in the development and refinement of clinical assessment instruments. *Psychological Assessment*, *7*(3), 286.

Gadow, K. D., & Sprafkin, J. (1997). *Child symptom inventory 4: CSI*. Checkmate Plus Stony Brook, NY.
